# The Main Dimensions of Sport Personality Traits: A Lexical Approach

**DOI:** 10.3389/fpsyg.2020.02211

**Published:** 2020-09-23

**Authors:** Reinout E. De Vries

**Affiliations:** Department of Experimental and Applied Psychology/Institute of Brain and Behavior Amsterdam, Vrije Universiteit Amsterdam, Amsterdam, Netherlands

**Keywords:** sport personality traits, lexical study, HEXACO, Big Five, sport and leisure activities

## Abstract

To uncover the main dimensions of sport personality traits, a lexical study was conducted. In the first two phases, 321 adjectives denoting the way somebody practices sports were selected. In the third phase, 555 respondents self-rated the adjectives. Congruence analyses provided evidence of six factors, five of which are sport personality trait factors (friendly fairness, resilience, drive, perfectionism, and inventiveness) plus one physical individual difference factor (agility). Marker scales from the sport personality trait factors show convergent correlations with the generic HEXACO personality obtained years earlier. Furthermore, meaningful relations with the six most frequently practiced sport and leisure activities were observed. Contextualized sport personality trait factors can be useful in research on sport preferences, sport behaviors, and sport outcomes.

## Introduction

Recently, there has been a proliferation of personality concepts that are specifically used in sport contexts, such as sport anxiety (Smith et al., 2006), mental toughness (Gucciardi, 2012), resilience (Sarkar and Fletcher, 2014), competitiveness (Gill and Deeter, 1988; Gill et al., 1988), moral disengagement (Boardley and Kavussanu, 2007), pro- and antisocial behaviors (Kavussanu and Boardley, 2009), aggressiveness (Maxwell and Moores, 2007), perfectionism (Gotwals et al., 2010), and (sport-specific) creativity (Memmert et al., 2010). There is a lack of research, however, that integrates the plethora of personality concepts in sport contexts and that offers a framework of the main sport personality traits. Here, the findings of a lexical study to uncover the main dimensions of sport personality traits are reported. Furthermore, this study investigates the relations between these dimensions of sport personality traits and personality – measured using the HEXACO model of personality (Lee and Ashton, 2004; Ashton et al., 2014) – and six frequently practiced sports and leisure activities.

### Sport Personality Traits

Research on sport (personality) traits^1^ can be roughly divided into two categories: research on personality *and* sports and research on personality *in* sports. The first, a “generic traits approach,” mainly studies relations between personality – broadly defined – and sport preference, physical activity, and sport performance (Eysenck et al., 1982; Gee et al., 2010; Woodman et al., 2010; Allen et al., 2013; Allen and Laborde, 2014). The second, a “contextualized traits approach,” mainly studies traits that are exhibited by people in the context of a sport setting. Researchers interested in the second approach are generally interested in specific traits that are exhibited while practicing or playing sports, such as sport anxiety (Smith et al., 2006), mental toughness (Gucciardi, 2012), resilience (Sarkar and Fletcher, 2014), aggressiveness (Maxwell and Moores, 2007), perfectionism (Gotwals et al., 2010), and moral disengagement (Boardley and Kavussanu, 2007).

#### Generic Traits Approach

Research on personality *and* sports can be subdivided into three distinct – but often overlapping – themes, i.e., research on (1) personality and sport preference (i.e., interest in particular sports), (2) personality and physical activity (including the developmental effects of physical activity and sport participation on personality), and (3) personality and sport performance (including personality profiles of successful athletes).

Studies that investigate the direct relations between *personality and sport preference* (1) have found that people who have higher levels of arousal, sensation seeking, extraversion, openness to experience, and/or emotional stability are more likely to prefer – and participate in – risky sports, such as mountaineering, motorcycling, and scuba diving (Kerr and Svebak, 1989; Kerr, 1991; Jack and Ronan, 1998; Tok, 2011). Furthermore, people participating in contact sports (e.g., soccer) are found to have higher levels of aggression than people participating in non-contact sports (e.g., volleyball; Lemieux et al., 2002). Some evidence is also available that people who participate in team sports (e.g., hockey) have, on average, somewhat higher levels of extraversion than people participating in individual sports (e.g., athletics; Eagleton et al., 2007), but this relation has not been sustained when comparing soccer and basketball to track and field (Steca et al., 2018). That is, the findings offer some support for a person–sport fit (P-S fit) hypothesis, which maintains that people who select sports that fit their personality are more likely to enjoy them, to be engaged in them, and to show less attrition.

With respect to *personality and physical activity* (2), meta-analyses have offered support for positive relations between extraversion, conscientiousness, and emotional stability (i.e., reversed neuroticism) and physical activity (Rhodes and Smith, 2006; Wilson and Dishman, 2015). A recent large-scale longitudinal Australian study (*N* = 10,227) shows positive longitudinal relations between conscientiousness and openness to experience on the one hand and physical activity on the other hand (Allen et al., 2017). When investigating the effects of physical activity on personality, longitudinal studies find significant positive associations between physical activity/sport participation and mean-level personality changes in extraversion and conscientiousness/persistence in two studies (Stephan et al., 2014; Allen et al., 2015), but not in the previously mentioned large-scale longitudinal study (Allen et al., 2017). Consequently, conscientiousness and extraversion appear – so far – to be the most consistent correlates of physical activity across different studies.

With respect to *personality and sport performance* (3), studies most often compare athletes on different (low to high) competition levels on personality variables. These studies consistently show positive relations between agreeableness, conscientiousness, and emotional stability and athletic performance (Kirkcaldy, 1982; Allen et al., 2011; Steca et al., 2018). Some of the personality–sport performance relations may be sport-specific. For instance, sensation seeking (i.e., high levels of extraversion and openness to experience and low levels of emotionality; De Vries et al., 2009c) is found to be positively related to performance among high-risk – but not among low-risk – free divers (Baretta et al., 2017). In general, the generic traits approach seems to offer support for the assumption that people prefer sports that “fit” their personality, that people with higher levels of conscientiousness and extraversion are more physically active, and that those with higher levels of conscientiousness, agreeableness, and emotional stability have more athletic success.

#### Contextualized Traits Approach

With respect to personality *in* sports, although scholars have long been interested in sport-contextualized traits, there is a lack of integration of generic and contextualized trait approaches in sports research. Contextualized traits are operationalized by either instructing respondents to keep a certain context in mind when answering items (instructional contextualization), by adding a tag that specifies the context to the items (tagged contextualization), or by completely rewriting the item to match the context (full contextualization) (Holtrop et al., 2014). Because contextualized items specify the situation, respondents are less likely to vary in the frame of reference when responding to an item, reducing both between-person variability and within-person inconsistencies, which, in turn, results in higher predictive validities when compared to generic constructs (Lievens et al., 2008).

Several contextualized sports trait measures have been constructed and used in a sports context although few are explicitly contextualized based on generic trait measures (Stoeber and Madigan, 2016). However, most contextualized sports traits show face resemblance and/or empirically correlate with similar generic traits. For instance, the sport anxiety scale (Smith et al., 2006), which measures somatic anxiety, worry, and concentration disruption in sport players, is found to be positively related to generic trait anxiety (Smith et al., 1990). Both mental toughness and resilience, two constructs that are often used in sports contexts, are found to be positively related to extraversion and negatively to neuroticism (Campbell-Sills et al., 2006; Horsburgh et al., 2009). Sport perfectionism, which is operationalized using the Sport Multidimensional Perfectionism Scale (Dunn et al., 2006), is found to be related to generic (non-contextualized) measures of perfectionism (Gotwals et al., 2010), which, in turn, are generally operationalized as a facet of conscientiousness (e.g., Lee and Ashton, 2004). Competitive anger and aggression in sports (Maxwell and Moores, 2007) are found to be related to physical aggression (Buss and Perry, 1992), which, in turn, is negatively related to agreeableness (Egan and Campbell, 2009). Furthermore, pro- and antisocial behaviors (Kavussanu and Boardley, 2009) and moral disengagement (Boardley and Kavussanu, 2007) are recent constructs in sports that involve volitional behaviors intended to help others (prosocial), harm others (antisocial), or breach moral standards (moral disengagement). These behaviors most closely resemble the generic honesty–humility construct (Ashton et al., 2014), which pits prosocial self-effacing behaviors intended to benefit others against antisocial self-enhancing behaviors intended to benefit the self.

Although the sports literature offers a rich palette of different – for sports – contextualized instruments, the main problem with the contextualized traits approach is that none of the studies can provide a definite answer to the question of which sport trait dimensions are really “fundamental,” which ones are not, and which traits that are fundamental have possibly not even been considered so far. That is, a framework to capture the main sport personality trait dimensions is lacking. This situation somewhat resembles the situation in personality psychology before the advent of the Big Five model (Goldberg, 1990). Until the advent of the Big Five, researchers derived their own models, which they based on theoretical (e.g., Eysenck and Eysenck, 1987) and/or empirical (e.g., the MMPI; Butcher et al., 1989) considerations. The so-called lexical paradigm ended this proliferation of personality models by proposing a standard, lexical method to arrive at a single unifying personality framework (Galton, 1884; Goldberg, 1990). As in generic lexical personality research, instead of relying on theoretical and/or empirical considerations, in this study, the lexical method is used to uncover the main sport personality trait dimensions.

### The Lexical Paradigm

According to the lexical paradigm, people describe their own and others’ personalities by using words that have subsequently become encoded in dictionaries. By following a standard procedure to extract the most common trait-related words from sufficiently large dictionaries and by asking respondents to indicate the extent to which these words represent their – or an acquaintance’s – personality, researchers across the world have been able to arrive at a near consensus about the main dimensions of personality. Based on studies that use the lexical paradigm, most personality psychologists ascribe to either five (Big Five; Goldberg, 1990) or six (HEXACO; Ashton et al., 2004; Lee and Ashton, 2004) dimensions of personality.

The lexical approach has been successfully applied to several psychology domains, such as values (Aavik and Allik, 2002; Renner, 2003), emotions (Clore et al., 1987; Storm and Storm, 1987), interpersonal interactions (Saucier, 1992; De Raad, 1995), and communication styles (De Vries et al., 2009b), but it has not yet been applied to the sport psychology domain. As noted above, such a lexical study may provide an integration of the sports personality literature by offering a framework of the most important sport trait dimensions.

### The Current Study

The aim of our study is to uncover the main sport trait dimensions and to find out how these sport traits relate to generic (HEXACO) personality traits and to sport and leisure activities. In line with other lexical research (e.g., De Vries et al., 2009b), this lexical study on sport traits was conducted in three phases. In the first phase, a preliminary selection of adjectives that pertained to ways of practicing sports was made. In the second phase, a further reduction of the list of adjectives was made based on a panel of experts. In the third and final phase, self-ratings were obtained on the list of adjectives selected in the previous phase using an internet panel. Furthermore, because members of this internet panel provided HEXACO personality data 3 or 7 years beforehand, it is possible to relate the dimensions of sport traits to the HEXACO personality domain scales. Additionally, information on sports-related behaviors was collected (e.g., sport and leisure activities), which allowed an investigation of the extent to which people who practice certain sports differ on sport traits from people who do not practice them. A link to the data from all three phases can be found in Supplementary Material.

## Materials and Methods

### Phase 1

In the first preliminary selection phase, three people evaluated a grand list of 7918 commonly used adjectives on whether these adjectives pertained to sport personality traits or not. A full description of how these adjectives were preselected is described in De Vries et al. (2009b, p. 183). The list of adjectives was rated once by three psychologists with expertise in personality and/or sports (two men, one woman), who were instructed to rate each adjective on whether it *reflects the way somebody practices a sport*. Using a three-point scale, raters were instructed to give the word a score of “2” if “the adjective refers to the way a person practices sports. That is, the adjective describes a trait that is relevant to practicing sports and/or describes behavior that is actually displayed while practicing sports.” They were instructed to give the word a score of “1” if “in doubt whether or not the adjective refers to a trait that is relevant to practicing sports and/or the way someone practices sports.” Finally, they were instructed to give the word a score of “0” if (a) the adjective does not refer to a trait that is relevant to practicing sports and/or the way someone practices sports, (b) the adjective does relate to sports or the public in sports but not to how someone practices sports, (c) the adjective does relate to the practice of other activities but does not give an adequate description of how someone practices sports, (d) the adjective is purely evaluative (like “good” and “bad”) but does not offer an adequate content description of how someone practices sports, and e) the adjective is unknown or very unusual or its meaning is vague or ambiguous. Furthermore, we offered the following tip: When unsure what score to give, the raters were asked to imagine whether a sports commentator could use the word to describe how somebody practices sports.

The intraclass correlation coefficients for this phase were 0.37 (ICC1,*k*) and 0.64 (ICC2,*k*), which can be considered acceptable for this first preliminary phase. The scores on each of the adjectives from the three raters were added to arrive at a total cumulative score, which ranged from 0 to 6. Adjectives with a score greater than or equal to 3 were retained, which resulted in 1113 words to be used in the next, more extensive, selection phase.

### Phase 2

In the second selection phase, the 1113 adjectives were rated by 14 expert raters (9 men, 5 women; *M*_*age*_ = 29.8, *SD*_*age*_ = 9.1) with a degree in sports and/or psychology. Eight raters rated the adjectives in alphabetical order; six raters rated the adjectives in reversed alphabetical order. Raters were instructed to rate each adjective on the extent to which it is related to *the way somebody practices sport*. Furthermore, raters were instructed to think about the following sentence when rating the adjective: “If s/he practices sports, s/he behaves in a…way” and to rate the adjective with a score between 1 and 5 on the prototypicality of the adjective for the way somebody practices sport, in which “5” stands for “provides a very clear picture of how somebody practices sports;” “4” for “provides a clear picture of how somebody practices sports;” “3” for “provides a vague picture of how somebody practices sports or the adjective is somewhat unusual, unfamiliar, difficult, or ambiguous;” “2” for “provides an unclear picture of how somebody practices sports or the adjective is unusual, unfamiliar, difficult, or ambiguous;” and “1” for “provides no or a very unclear picture of how somebody practices sports or the adjective is very unusual, unfamiliar, difficult, or ambiguous.”

The intraclass correlation coefficients for this second phase were 0.28 (ICC1,*k*) and 0.84 (ICC2,*k*). The scores on each of the adjectives from the 14 raters were averaged, and for the next phase, only adjectives with scores of 3 or higher were retained. The 321 adjectives that fulfilled this criterion and that were selected for phase 3 are reported in Supplementary Table S1 together with the mean prototypicality ratings (plus standard deviations) of each of the adjectives.

### Phase 3

In the third phase, all adjectives that were retained after phase two were used to obtain self-ratings on sport traits. This data was combined with personality and sport and leisure activities data collected earlier using the same sample.

#### Sample and Procedure

To obtain the sport traits data, an ISO-certified population-representative internet panel was used (i.e., ISO 26362, which confirms that the panel satisfies the quality requirements for panels for social science, market, and opinion research). Part of this panel has been used in previous research (e.g., De Vries and Van Kampen, 2010; De Vries et al., 2013; De Vries and Van Gelder, 2015) in which – among others – the HEXACO Personality Inventory–Revised (Lee and Ashton, 2004; De Vries et al., 2009a) was filled out years earlier. Of the 1672 panel members approached who were part of one of the earlier studies, a sample of 609 respondents (36.4%) completely filled out the sport traits adjectives questionnaire. However, probably due to the length of the questionnaire, the sample contained a substantial number of straight-lining responses. As noted above, the main part of the questionnaire contained 321 adjectives on which the respondents had to provide self-ratings. We removed 54 respondents who exhibited straight-lining responses [i.e., who gave the same response on more than 310 out of 321 (>96.5% straight-lining responses) adjectives], retaining a final usable sample of 555 respondents (48.3% women). The mean age – at the time when the respondents filled out the sport personality questionnaire – was 50.1 years (*SD* = 14.7). In addition, 20.0% of the sample had a low educational level (e.g., no or low level of secondary and/or vocational education), 41.8% a medium level of education (e.g., medium or high level of secondary education and/or low level of tertiary education), and 38.2% had a high level of education (e.g., medium or high level of tertiary education).

### Instruments

#### Sport Traits Adjectives

The 321 adjectives from phase 2 were provided in a completely random order to the respondents. An instructional contextualization was used, in which respondents were instructed to indicate the extent to which they showed the sport behaviors when comparing themselves to others. The leading instructional contextualization sentence that was displayed with each adjective was “When playing sports, I behave in a…way” in which the dots (…) were to be replaced with the adjective. The respondents could respond to the statement with 1 = much less than others, 2 = less than others, 3 = average when compared to others, 4 = more than others, and 5 = much more than others. This response format was chosen because (1) for ease of use, it made it possible to use the same response options for all items, (2) it ensured a better approximation of a normal distribution in responses (i.e., lower skewness because of a mean of responses around the midpoint of the scale), and (3) research has shown that agree–disagree response options may violate the assumed monotonic relation between traits and responses, which, as a consequence, lowers the quality of responses when compared with response options that are more aligned with the underlying scale of interest – in this case, how often the respondents behaved in a certain way when comparing themselves to others (Saris et al., 2010).

#### Sport and Leisure Activities

Apart from the sport personality adjectives, we also asked respondents to indicate which sports and leisure activities they practiced (up to five sports or leisure activities maximally) and how much time they spent on each of these sports/leisure activities. The list of sports and leisure activities was obtained from the Dutch “list of sports” Wikipedia web page (downloaded November 2011)^2^, containing 261 sports and leisure activities, distributed among 19 categories, such as athletics, ball sports, mind sports, power sports, motor sports, water sports, fight sports, walking sports, winter sports, extreme sports, etc. The 10 sports/leisure activities most practiced by respondents were fitness (149 times mentioned), running (131), soccer (122), solving puzzles (118), swimming (103), tennis (98), doing sudoku (73), volleyball (70), badminton (64), and playing chess (40). All of the respondents practiced sports, but 168 respondents indicated they had not practiced their number one sports or leisure activities during the last year (although they had practiced other sports). The remaining 387 respondents indicated that they had practiced their favorite sports/leisure activity on average 1.99 days a week (*SD* = 1.56) with a mean of 2:10 h (*SD* = 2:31 h) per week.

#### HEXACO-PI-R

Of the 555 respondents, 449 respondents had filled out the Dutch HEXACO Personality Inventory–Revised (Lee and Ashton, 2004; De Vries et al., 2009a) either 7 (*n* = 178) or 3 years earlier (*n* = 271). The HEXACO-PI-R consists of 200 items of which 192 pertain to the six main personality domains – honesty–humility, emotionality, extraversion, agreeableness, conscientiousness, and openness to experience – and eight items pertain to the interstitial facet altruism. An additional eight proactivity items (De Vries et al., 2016b), pertaining to an interstitial facet that is associated with extraversion, conscientiousness, and openness to experience, were filled out by the latter *n* = 271 sample. The HEXACO items were answered on a traditional 1–5 (strongly disagree–strongly agree) scale. In this sample of 449 respondents, the alpha reliabilities of the HEXACO domain scales were 0.91 (honesty–humility), 0.88 (emotionality), 0.89 (extraversion), 0.89 (agreeableness), 0.84 (conscientiousness), and 0.86 (openness to experience).

#### Analyses

Within-person standardization (ipsatization) was carried out on the 321 sport personality adjectives to remove variance associated with individual answering tendencies (e.g., acquiescence, nay-saying, extreme answering). Subsequently, principal component analyses (PCAs) with varimax rotation were conducted on the ipsatized data. To determine the optimal factor solution, multiple bootstrapped (*k* = 500) within-sample congruence coefficients using a randomly split between-subjects PCA with orthogonal Procrustes rotation were obtained. Procrustes analysis was used because it is shown to provide robust estimates of factor comparability (Paunonen et al., 1992; McCrae et al., 1996) and because other methods to determine the optimal number of components in large item sets often yield too many components. As described below, Procrustes analyses offered support for not more than six PCs. In contrast, parallel analyses (Horn, 1965; O’Connor, 2000) yielded twice the number of components (e.g., 12 PCs) in the actual data that had higher eigenvalues than the 95th percentile of the eigenvalues of randomly generated data.

In the Procrustes analyses, for each factor solution (from two to eight factors), the sample of 555 respondents was randomly split in half 500 times. Subsequently, a PCA was conducted on each of the two randomly obtained subsamples, and targeted orthogonal rotation was performed to compare the PCs in the 500 solutions. Subsequently, the congruence coefficients for the components were averaged to provide a single estimate of the factor congruence.

### Phase 3 Results

#### Factor Congruence

The results of the within-sample randomly split between-subjects PCAs with orthogonal Procrustes rotation on two to eight factor solutions are reported in Table 1. Based on a simulation study, Paunonen (1997) argues that researchers who find congruence coefficients in excess of 0.73 can be reasonably confident that the level of fit is not simply due to sampling error. Furthermore, using real data, Paunonen et al. (1992) find that a congruence coefficient level of 0.75 is approximately the level at which observed congruence coefficients are significantly higher (at *p* < 0.05) than the mean of 1000 randomly generated congruence coefficients. Consequently, and in line with De Vries et al. (2009b), a cutoff level of 0.75 for the congruence coefficients was employed. The results in Table 1 show that only the two and four PC solutions fully correspond to this criterion and that, in PC solutions three, five, and six, one of the PCs had congruence coefficients lower than 0.75. A notable result is that in both PC solutions four and six, the – respectively – third and fifth congruence coefficients do exceed the 0.75 cutoff level, whereas the third and fifth congruence coefficients of the – respectively – third and fifth PC solutions do not. When looking at the content of the respective PC solutions, the sixth component of the sixth PC solution shows face resemblance to the openness to experience factor in personality research. Thus, even though the sixth component does not conform to the initial 0.75 cutoff, suggesting that the cross-sample stability of this factor may need further empirical evidence, for further analyses it was decided to focus on this six-factor solution.

**TABLE 1:** Mean congruence coefficients of 500 within-sample randomly split between-subjects PCAs of 321 ipsatized sport adjectives (*N* = 555) using orthogonal Procrustes rotation of up to eight components.

Components	1st	2nd	3rd	4th	5th	6th	7th	8th	Mean
2 PC solution	0.96	0.94							0.95
3 PC solution	0.94	0.92	0.73						0.86
4 PC solution	0.94	0.93	0.89	0.84					0.90
5 PC solution	0.93	0.90	0.84	0.80	0.69				0.83
6 PC solution	0.94	0.92	0.86	0.84	0.80	0.68			0.84
7 PC solution	0.93	0.92	0.86	0.83	0.78	0.67	0.58		0.80
8 PC solution	0.93	0.92	0.86	0.83	0.77	0.69	0.64	0.54	0.77

#### Factor Tree

**FIGURE 1:**
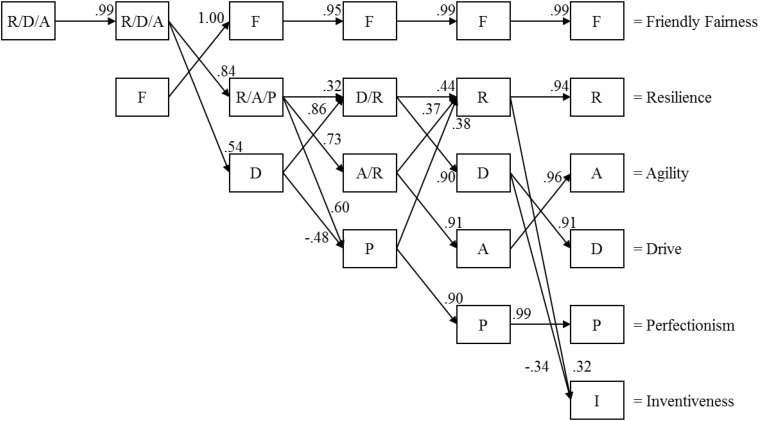
Factor tree with Pearson’s correlations > 0.30 of the 1- to 6-PC solutions of the 321 ipsatized sport adjectives (*N* = 555).

A PCA-based factor tree of the first six components is displayed in Figure 1. For each of the 1- to 6-PC solutions, the components were saved as variables and were correlated with components from subsequent PC solutions. The adjectives of the six PC solutions are shown in Table 2 together with their provisional names, e.g., friendly fairness, resilience, agility, drive, perfectionism, and inventiveness. As the factor tree shows, the first component of the 1-PC solution is strongly (*r* = 0.99) correlated with the first component of the 2-PC solution, being mainly associated with the – later named – factor resilience, but also containing some variance associated with the later called agility and drive components. The first 30 adjectives in the 1- and 2-PC solutions have negative loadings on this component and are associated mainly with sluggishness, tiredness, lack of motivation, and uncertainty [e.g., sluggish (−0.69), exhausted (−0.67), and uncertain (−0.66) in the 1-PC solution and sluggish (−0.73), uncertain (−0.69), and slow (−0.68) in the 2-PC solution]. The second component of the 2-PC solution – the provisionally called friendly fairness component – remains highly similar to the first component in subsequent PC solutions. Examples of adjectives and loadings are found in Table 2.

**TABLE 2:** Highest loading adjectives in the six varimax rotated PC solutions.

**1. Friendly Fairness (5.84%)**
Good-humored (0.56), kind (0.55), friendly (0.55), social (0.52), fair (0.52), tolerant (0.52), very hard (−0.51), merciless (−0.51), solitary (0.51), hard as nails (−0.50), rough (−0.49), destructive (−0.49), helpful (0.49), decent (0.47), hard (−0.47), hard as stone (−0.47), attentive (0.47), rock-hard (−0.46), just (0.45), hostile (−0.44), venomous (−0.44), brutish (−0.44), enraged (−0.44), honest (0.44), cheerful (0.44), merry (0.43), cooperative (0.43), harsh (−0.43), antagonistic (−0.43), harmonious (0.43).
**2. Resilience (4.58%)**
Nervous (−0.64), uncertain (−0.64), stressed (−0.63), jittery (−0.62), hesitating (−0.61), strained (−0.58), cramped (−0.55), tired (−0.54), exhausted (−0.53), weary (−0.51), worn out (−0.51), defeated (−0.50), frustrated (−0.49), clumsy (−0.49), self-assured (0.48), run down (−0.47), restless (−0.46), injured (−0.46), inconsistent (−0.45), battle weary (−0.44), stiff (−0.44), forced (−0.43), slow (−0.43), demotivated (−0.42), amateurish (−0.41), hurried (−0.41), confident (0.40), sloppy (−0.40), cautious (−0.39), restrained (−0.38).
**3. Agility (3.05%)**
Supple (0.52), fast (0.52), swift as an arrow (0.51), quick (0.51), lithe (0.50), graceful (0.50), mobile (0.50), lightning fast (0.49), acrobatic (0.45), sluggish (−0.44), trained (0.41), dynamic (0.40), inexhaustible (0.39), maneuverable (0.39), fluent (0.37), fleet-footed (0.37), talented (0.37), agile (0.37), effortless (0.36), tireless (0.35), elegant (0.35), excellent (0.35), adept (0.34), flashy (0.34), vital (0.34), cast iron (0.33), energetic (0.32), toned (0.30).
**4. Drive (2.94%)**
Fanatical (0.47), unmotivated (−0.44), driven (0.44), enthusiastic (0.44), ardent (0.43), passive (−0.43), pugnacious (0.42), assertive (0.42), passionate (0.41), eager (0.40), animated (0.38), temperamental (0.37), combative (0.37), fiery (0.35), motivated (0.35), intense (0.34), truculent (0.33), hot-blooded (0.33), strong-willed (0.32), exuberant (0.32), zealous (0.32), impassioned (0.31), persevering (0.31), spirited (0.31), elated (0.30), non-chalant (−0.30).
**5. Perfectionism (2.42%)**
Meticulous (0.46), accurate (0.44), perfectionistic (0.43), thorough (0.40), precise (0.37), careful (0.36), tactical (0.36), skilled (0.35), deadly serious (0.35), efficient (0.35), capable (0.35), systematical (0.35), proficient (0.34), well thought out (0.34), well considered (0.33), specialist (0.32), alert (0.31), overconfident (−0.31), targeted (0.30).
**6. Inventiveness (1.64%)**
Full of fantasy (0.44), creative (0.44), imaginative (0.42), inventive (0.39), resourceful (0.38), innovative (0.37), surprising (0.36), smart (0.30), serious (−0.30).

**No more than the 30 highest loading adjectives with absolute loadings ≥ 0.30 are reported. The original Dutch words and additional descriptives and loadings are reported in Supplementary Table S1. Percentage of explained variance of PCs is noted in parentheses.**

The second component of the 2-PC solution is split up in component two, which contains variance associated with resilience, agility, and perfectionism, and in component three (mainly drive) of the 3-PC solution. In the 4- and 5-PC solutions, the variance associated with these two components is distributed and redistributed in a complex way among the different components until, in the 5-PC solution – apart from friendly fairness – relatively stable PCs, e.g., resilience, drive, agility, and perfectionism, emerge. Finally, in the 6-PC solution, inventiveness appears as a by-and-large new component.

Apart from the 6-PC solution, analyses were performed on additional PC solutions to see whether there was any evidence for robust interpretable factors beyond the first six. The analyses are reported in Supplementary Table S2. Beyond six, the components are relatively small, difficult to interpret, and unstable. Compared to these, the components from the 6-PC solution are highly robust and remain present in the PC solutions that follow. Consequently, when evaluating the solutions in terms of content and robustness, the 6-PC solution appears to be most optimal in the current data set.

#### Sport Personality Marker Scales and Personality

Marker scales were constructed based on the highest loading adjectives (after reverse-coding negative-loading adjectives) of the six sport trait dimensions reported in Table 2. Apart from the sixth component, inventiveness, all alpha reliabilities of the marker scales are higher than 0.90 (see Table 3), and except for friendly fairness, means are less than one standard deviation from the midpoint (3). The relations with the background variables gender, age, and educational level (1 = low to 3 = high) are generally not very strong. As might be expected, age is negatively related to agility (*r* = −0.25, *p* < 0.01) and all relations with gender and educational level are lower than – or equal to – the 0.20 level.

**TABLE 3:** Descriptives (alpha reliabilities on diagonal) and correlations of sport personality marker scales with background variables, HEXACO personality, and sport personality PCs.

		Background			Personality						Sport personality marker scales					
				
		Gender	Age	Education	H	E	X	A	C	O	F	R	A	D	P	I
1.	Gender (F = 0; M = 1)	–														
2.	Age	0.17	–													
3.	Education (low = 1, high = 3)	−0.06	−0.25	–												
**Personality**																
4.	Honesty–humility	−0.14	0.27	−0.03	*0.91*											
5.	Emotionality	−0.44	−0.01	−0.02	0.11	*0.88*										
6.	Extraversion	0.08	0.04	0.04	−0.02	−0.22	*0.89*									
7.	Agreeableness	0.08	0.08	−0.02	0.37	−0.16	0.14	*89*								
8.	Conscientiousness	−0.01	0.03	0.05	0.16	−0.13	0.27	0.11	*0.84*							
9.	Openness to experience	0.02	0.11	0.27	−0.04	−0.05	0.18	0.06	0.10	*0.86*						
**Sport personality marker scales**																
10.	Friendly fairness	−0.10	0.16	0.03	***0.36***	0.09	0.10	*0.21*	0.13	0.15	*0.93*					
11.	Resilience	0.11	0.05	−0.02	0.09	−*0.23*	***0.35***	0.13	0.20	0.09	0.28	*0.93*				
12.	Agility	0.18	−0.25	0.05	−**0.29**	−0.22	0.27	0.01	0.05	0.11	−0.22	0.35	*0.95*			
13.	Drive	0.20	−0.13	0.02	−0.16	−0.18	**0.29**	0.02	0.11	0.11	−0.10	0.32	0.73	*0.94*		
14.	Perfectionism	0.17	−0.13	0.12	−0.12	−0.19	0.14	0.07	***0.23***	0.17	0.14	0.29	0.61	0.65	*0.91*	
15.	Inventiveness	0.17	−0.09	0.05	−0.20	−0.18	0.22	0.09	−0.01	***0.29***	−0.06	0.18	0.62	0.58	0.60	*0.80*
**Sport personality PCs**																
16.	Friendly fairness	−0.15	0.19	0.00	***0.40***	0.12	0.08	*0.22*	0.08	0.08	***0.86***	0.24	−0.27	−0.20	−0.06	−0.13
17.	Resilience	0.29	0.02	−0.01	−0.05	−***0.42***	*0.33*	0.09	0.16	0.12	0.05	***0.76***	0.36	0.39	0.36	0.27
18.	Agility	−0.08	−0.21	0.01	−0.10	0.00	0.11	0.07	0.02	−0.04	−0.06	0.29	***0.63***	0.14	0.14	0.13
19.	Drive	0.01	0.00	−0.04	0.02	0.07	**0.23**	−0.03	0.06	0.02	0.02	0.16	0.33	***0.73***	0.25	0.24
20.	Perfectionism	0.00	0.02	0.14	0.06	−0.05	−0.14	0.00	***0.25***	0.03	0.14	0.08	0.07	0.09	***0.61***	0.13
21.	Inventiveness	0.11	0.03	0.07	−0.12	−0.13	0.21	0.06	−0.03	***0.27***	0.04	0.03	0.22	0.10	0.22	***0.65***

		Gender	Age	Education	H	E	X	A	C	O	F	R	A	D	P	I

Mean		0.52	50.05	2.18	3.71	3.08	3.32	3.06	3.48	3.21	3.52	3.29	2.76	3.02	3.10	2.98
Standard deviation		0.50	14.72	0.74	0.47	0.44	0.45	0.43	0.38	0.45	0.42	0.46	0.50	0.46	0.41	0.42

**HEXACO (N = 449) personality scales are obtained 3 or 7 years earlier; all other analyses are based on concurrent data with N = 555. For the HEXACO correlations, if r > 0.12, p < 0.01. Otherwise, if r > 0.10, p < 0.01. In the three 6 × 6 blocks in the table, highest block-wise correlations > 0.20 in rows are bolded; highest block-wise correlations in columns are italicized.**

To establish whether the marker scales represent the PCs well, in Table 3, the correlations between the marker scales and the original PCs are reported. All of the convergent correlations are > 0.60, and none of the discriminant correlations is > 0.40. The marker scales friendly fairness, resilience, and drive most closely represent the PCs from which they are derived, all with convergent correlations > 0.70.

Finally, both the sport personality marker scales and the PCs were correlated with HEXACO personality. HEXACO personality data (*N* = 449) were obtained 7 (*n* = 178) or 3 (*n* = 271) years earlier, so the correlations reported are likely to underestimate the correlations that would be found if the data were obtained concurrently. The correlations between the HEXACO personality scales and the sport personality marker scales on the one hand and the sport personality PCs on the other are reasonably similar. That is, the two blocks of correlations correlate at 0.75 (*p* < 0.01). However, the pattern of high and low correlations of the HEXACO personality scales with the PCs is somewhat more extreme than with the marker scales.

The results show that friendly fairness is most closely associated with honesty–humility and also somewhat with agreeableness. Resilience is associated with both emotionality and extraversion, whereas drive is associated exclusively with extraversion. As might be expected, perfectionism is most closely associated with conscientiousness, and inventiveness is most closely associated with openness to experience. The most important outlier in the analyses is agility, at least in relation to the sport personality PCs, in which agility does not show any absolute correlations with HEXACO personality surpassing the 0.11 level. Interestingly, the relations between HEXACO personality and the agility marker scale are somewhat higher with the most important predictors low honesty–humility, low emotionality, and high extraversion. However, when looking at the adjectives of agility, these do not seem to reflect sport personality proper, but rather individual differences in physical capacities. The place of agility in the sport personality framework is discussed in the general discussion and conclusion sections.

In the Supplementary Material, the correlations between the six sport personality marker scales and all the HEXACO facets are provided (Supplementary Table S3). The results are generally in line with the HEXACO domain scales although, within HEXACO domains, some differential relations between facets and sport personality marker scales are observed.

#### Sport Traits and Sport and Leisure Activities

In Tables 4, 5, the results of the logistic regressions of the six most frequently practiced sports and leisure activities on the background variables gender, age, education level, and the six sport personality marker scales are shown^3^. In all the models, the omnibus χ^2^-test of significance for the entire model containing all nine variables is significant. The results indicate that practicing fitness is associated with lower levels of inventiveness, practicing running is associated with higher levels of drive, and playing soccer is associated with higher levels of perfectionism (Table 4). The only leisure activity in the analyses, solving puzzles, is unrelated to any of the sport traits. This is also the case for swimming, which is unrelated to any of the sport traits. Practicing tennis, however, is associated with higher levels of agility (Table 5). To check whether the sport traits still predict sport activity when entered together with the HEXACO personality variables, logistic regression analyses were run in which the background and HEXACO variables were entered first and the sport personality scales last. The results (with a somewhat lower *N* = 449), which are presented in Supplementary Tables S4.1–S4.6, are very similar for the sport traits^4^ and show some additional effects of personality variables on the sport and leisure activities (e.g., extraversion is negatively associated with the leisure activity of solving puzzles).

**TABLE 4:** Logistic regression of the most practiced sports and leisure activities on background and sport personality traits (*N* = 555).

	**Fitness (*n* = 149)**			**Running (*n* = 131)**			**Soccer (*n* = 122)**		
			
	**Odds**	**95% CI**	***d***	**Odds**	**95% CI**	***d***	**Odds**	**95% CI**	***d***
**Background**									
1. Gender (0 = F, 1 = M)	0.88	(0.59–1.32)	−0.07	1.61*	(1.04–2.48)	0.26	11.91**	(6.17–23.01)	1.37
2. Age	0.98**	(0.96–0.99)	−0.01	0.98*	(0.97–1.00)	−0.01	0.99	(0.97–1.01)	−0.01
3. Education (1 = Lo thru 3 = Hi)	1.20	(0.91–1.58)	0.10	1.15	(0.86–1.53)	0.08	0.48**	(0.34–0.67)	−0.41
**Sport personality traits**									
4. Friendly Fairness	1.38	(0.79–2.40)	0.18	0.94	(0.53–1.65)	−0.04	0.90	(0.46–1.75)	−0.06
5. Resilience	1.01	(0.62–1.66)	0.01	0.96	(0.55–1.68)	−0.02	0.69	(0.35–1.37)	−0.20
6. Agility	1.13	(0.57–2.25)	0.07	1.69	(0.82–3.48)	0.29	0.98	(0.43–2.23)	−0.01
7. Drive	1.34	(0.70–2.59)	0.16	2.09*	(1.03–4.23)	0.41	1.48	(0.68–3.22)	0.22
8. Perfectionism	0.89	(0.45–1.80)	−0.06	0.51	(0.23–1.12)	−0.37	4.69**	(1.92–11.47)	0.85
9. Inventiveness	0.39**	(0.20–0.75)	−0.52	1.14	(0.58–2.25)	0.07	1.05	(0.49–2.26)	0.03

Nagelkerke pseudo *R*^2^	6.8%			9.2%			37.1%		
χ^2^	26.71, *df* = 9, *p* < 0.01			35.03, *df* = 9, *p* < 0.01			153.58, *df* = 9, *p* < 0.01		

*p < 0.05; **p < 0.01.

**TABLE 5:** Logistic regression of the most practiced sports and leisure activities on background and sport personality traits (*N* = 555).

	**Solving puzzles (*n* = 118)**			**Swimming (*n* = 103)**			**Tennis (*n* = 98)**		
			
	**Odds**	**95% CI**	***d***	**Odds**	**95% CI**	***d***	**Odds**	**95% CI**	***d***
**Background**									
1. Gender (0 = F, 1 = M)	0.32**	(0.20–0.52)	−0.62	0.26**	(0.16–0.43)	−0.75	0.72	(0.45–1.16)	−0.18
2. Age	1.07**	(1.05–1.09)	0.04	1.00	(0.98–1.02)	0.00	1.01	(1.00–1.03)	0.01
3. Education (1 = Lo thru 3 = Hi)	0.96	(0.71–1.31)	−0.02	1.08	(0.79–1.48)	0.04	1.43*	(1.03–1.98)	0.20
**Sport personality traits**									
4. Friendly Fairness	1.12	(0.58–2.15)	0.06	1.45	(0.76–2.76)	0.20	1.43	(0.75–2.74)	0.20
5. Resilience	0.84	(0.48–1.46)	−0.10	1.10	(0.63–1.93)	0.05	0.61	(0.32–1.16)	−0.28
6. Agility	1.02	(0.46–2.26)	0.01	0.91	(0.41–2.00)	−0.05	4.05**	(1.74–9.42)	0.77
7. Drive	0.66	(0.30–1.43)	−0.23	0.91	(0.42–2.01)	−0.05	0.72	(0.32–1.63)	−0.18
8. Perfectionism	1.58	(0.69–3.60)	0.25	0.80	(0.35–1.82)	−0.12	1.50	(0.63–3.55)	0.22
9. Inventiveness	0.82	(0.38–1.75)	−0.11	1.40	(0.67–2.93)	0.19	0.86	(0.41–1.83)	−0.08

Nagelkerke pseudo *R*^2^	22.1%			11.2%			7.9%		
χ^2^	85.13, *df* = 9, *p* < 0.01			39.77, *df* = 9, *p* < 0.01			27.16, *df* = 9, *p* < 0.01		

*p < 0.05; **p < 0.01.

### Phase 3 Conclusion and Discussion

Congruence analyses provide evidence for the existence of six unique and clearly interpretable sport traits factors, named friendly fairness, resilience, agility, drive, perfectionism, and inventiveness. None of the additional factor solutions provides evidence of stable and well-interpretable extra factors. Both the sport traits marker scales and the PCs show consistent convergent correlations with generic HEXACO personality scales measured 3–7 years earlier with friendly fairness most strongly related to (generic) honesty–humility and agreeableness, resilience most strongly related to emotionality and extraversion, agility not consistently related to any of the sport personality scales, drive related mainly to extraversion, perfectionism to conscientiousness, and inventiveness to openness to experience. When relating the marker scales to the six most often practiced sports and leisure activities, practicing fitness is associated with lower levels of inventiveness, running with higher levels of drive, soccer with higher levels of perfectionism, and tennis with higher levels of agility.

## General Discussion and Conclusion

Using the lexical paradigm, this study provides a framework of the main sport personality trait dimensions, named friendly fairness, resilience, agility, drive, perfectionism, and inventiveness. These six independent dimensions appear to cover the most important concepts that people use when discussing how they behave when practicing sports. Five of the six dimensions uncovered in the lexical study not only relate logically to generic HEXACO personality dimensions, but also show strong face validity when compared to existing scales, such as moral disengagement (Boardley and Kavussanu, 2007), aggressiveness (Maxwell and Moores, 2007), and pro- and antisocial behaviors (Kavussanu and Boardley, 2009) (friendly fairness), resilience (Sarkar and Fletcher, 2014), mental toughness (Gucciardi, 2012), and (reversed) sport anxiety (Smith et al., 2006) (resilience), competitiveness (Gill and Deeter, 1988; Gill et al., 1988) (drive), perfectionism (Gotwals et al., 2010) (perfectionism), and sport-specific creativity (Memmert et al., 2010) (inventiveness). The only scale that seems to be an outlier in the analyses is the agility dimension. Agility, as found in the lexical research, pertains to physical instead of psychological individual differences, i.e., being (physically) supple, fast, lithe, graceful, mobile, and acrobatic, and can, thus, not be regarded as a proper personality trait. This factor emerges because in phases 1 and 2, no explicit instruction was provided to exclude purely physical behaviors or traits when rating the suitability of the adjectives to describe ways somebody practices sports. Consequently, five of the six dimensions seem to refer to individual differences in sport personality traits, and one dimension, agility, seems to refer to individual differences in physical sport capacities.

Apart from the logical relations with generic personality, the sport traits are found to be related to the actual practice of sports, possibly indicating the presence of P-S fit. P-S fit is a subset of the more general person-environment fit (P-E fit) concept (French et al., 1974; Caplan, 1987; Edwards and Rothbard, 1999). The P-E fit hypothesis – and, for that matter, the P-S fit hypothesis – maintains that people are attracted to specific environments (e.g., particular sports) that are commensurate with their personality and that people are more satisfied and perform better in environments/sports in which there is a good “fit” between their traits and environmental characteristics.

There are some limitations and caveats associated with this lexical study. First of all, without cross-cultural verification, it is impossible to confirm whether the dimensions uncovered in this study are replicated in other cultures. Although cross-cultural generic lexical personality studies generally align in their findings (e.g., Ashton et al., 2004; Saucier, 2009; De Raad et al., 2014), without further research, this simply cannot be affirmed for sport-contextualized personality. Second, this study was conducted among somewhat older participants (*M*_*age*_ = 50.1 years; *SD*_*age*_ = 14.7), and the sample consists of people who participate in a wide variety of sports – and leisure – activities (note, though, that all of the participants in this study practiced sports). The upside of this sample is (a) that it ensures a sufficient level of variance in the sport traits that are measured and (b) that it is possible to relate the sport trait dimensions to earlier collected generic personality ratings. However, a downside of this study is that it is only possible to relate the sport traits to five sport activities with a sufficient number of participants. Note that the inclusion of leisure activities, which are unrelated to the sport traits, also helps to establish the discriminant validity of the sport traits. Third, the relations between the sport personality marker scales and generic HEXACO personality are somewhat weaker than may be expected; i.e., absolute convergent correlations with the HEXACO-PI-R personality scales range between 0.23 and 0.42 for the sport personality PCs and marker scales (vs. correlations ranging between 0.49 and 0.78 between the HEXACO-PI personality scales and generic personality lexical marker scales measured concurrently; see De Vries et al., 2009a). Two important reasons for these somewhat weaker-than-expected relations may be that (a) HEXACO personality was measured 3– 7 years earlier, and (b) the list of adjectives was very lengthy, resulting in more error variance associated with careless responding. However, it may also be true that sport traits are less well aligned with generic personality traits or that people exhibit somewhat different traits in sport contexts. Future research may investigate whether a shorter – marker scale – sport traits instrument shows stronger relations when generic personality is measured concurrently.

Fourth, the relations between sport personality traits and the practiced sports and leisure activities are also relatively weak. Apart from the effect of survey length, another reason for these relatively weak findings may be that other variables, such as physical talent, parental interests and pressure, and the availability of sport and leisure opportunities in the childhood environment play a stronger role than personality (e.g., Ferreira et al., 2006). Future research may investigate whether individual differences in sport preferences play a role in sport and leisure activity choices, similar to the role vocational interests play in choosing and persisting in an academic major and a vocational choice (e.g., Rottinghaus et al., 2007; Allen and Robbins, 2008). An important difference between sport preference/interests and vocational interests is that the latter is more often an adult choice, whereas important sport- and leisure activity–related choices are often made in childhood.

All in all, the sport traits framework uncovered in this study may be regarded as a starting point for future research in the predictive validity of these factors for sport preference, behaviors exhibited in different sports, and sport performance. Although a selection of the top 9 or 10 adjectives for each sport personality factor – with instructional contextualization – can be used in future research for this purpose, the use of single adjectives may invite higher levels of social desirable responding than the use of questionnaire items (John and Robins, 1993; Wood and Wortman, 2012; De Vries et al., 2016a), and thus, it would be recommendable to create tagged or fully contextualized sentence items to cover the sport personality factors. Another suggestion would be to conduct a factor analysis on existing instruments that appear to cover a similar factor space, i.e., pro- and antisocial behavior (cf. friendly fairness), resilience, mental toughness, and/or sport anxiety (cf. resilience), competitiveness (cf. drive), perfectionism, and sport-related creativity (cf. inventiveness).

Follow-up research might also investigate whether such a sport traits questionnaire – based on either new or existing items – indeed adequately covers the same sport traits factor space as the lexically derived sport traits factors. Subsequently, the predictive validity of such a sport traits questionnaire can be compared to generic personality questionnaires. Because specific sport traits constructs are contextualized versions of generic personality constructs, the expectation is that sport traits offer incremental validity in the prediction of sport preferences, behaviors, and outcomes on top of generic personality questionnaires as has been found in other contextualization studies (e.g., Lievens et al., 2008; Shaffer and Postlethwaite, 2012; Holtrop et al., 2014)^5^. However, because sport preferences involve more activities than only behaviors exhibited while playing sports (e.g., socializing after the game), future research might investigate whether contextualization provides somewhat less incremental validity over generic personality traits in the prediction of sport preferences than in the prediction of sport behaviors and sport outcomes.

To conclude, using a lexical approach, this research shows that the sport personality traits factor space can be adequately described by six factors, one of which pertains to physical individual differences and five of which pertain to individual differences in sport personality traits. Furthermore, these sport traits are found to differentially relate to sport activities. This research is the first to offer an integrative framework of contextualized personality factors in a sport context using a lexical approach. This sport traits framework may help researchers to integrate knowledge based on different sport personality traits instruments (e.g., using meta-analyses), to become aware of research areas in sport traits that require further attention, and to further our knowledge on the potentially important effects of sport traits on sport preferences, sport behaviors, and sport outcomes.

## Data Availability Statement

All datasets generated for this study are included in the article/Supplementary Material.

## Ethics Statement

The studies involving human participants were reviewed and approved by the VCWE-2015-067 Vrije Universiteit Amsterdam. The participants provided their written informed consent to participate in this study.

## Author Contributions

REDV collected and analyzed the data and wrote the manuscript.

## Conflict of Interest

The HEXACO-PI-R is available for commercial purposes. A percentage of the profit from sales by the Vrije Universiteit Amsterdam is used to support the author’s research.

## Footnotes

1 In the remainder of the manuscript, the abbreviated “sport traits” term is used when referring to sport personality traits. Note that, throughout, “sport traits” refer to “broad contextualized sport personality traits”; that is, the sport traits are, on the one hand, regarded as broad traits (not facets) that cannot be subsumed under higher-order sport traits and, on the other hand, as contextualized in the sense that they are subsumed under even broader generic personality domains.

2 https://nl.wikipedia.org/w/index.php?title=Lijst_van_sporten

3 We chose only the six most frequently practiced sport and leisure activities (with occurrence rates > 15%) to prevent biases in the logistic regressions from occurring as a consequence of the use of rare events data (see King and Zeng, 2001).

4 Drive turns out to be only marginally significant (*p* = 0.06) in the prediction of running, probably because of its collinearity with extraversion, which is significantly related to running in the first step but not significant anymore when the sport personality scales (including drive) are added to the logistic regression equation.

5 Note that, in sports research, two levels of contextualization can be distinguished. Generic personality items are contextualized to a general sports context, and they are even further contextualized to specific sports (e.g., fitness, running, soccer, etc.). Whether this “second order” contextualization has additional incremental value in the prediction of criteria, may constitute another important area of research.
