# Corrigendum: The Main Dimensions of Sport Personality Traits: A Lexical Approach

**DOI:** 10.3389/fpsyg.2021.666906

**Published:** 2021-05-13

**Authors:** Reinout E. De Vries

**Affiliations:** Department of Experimental and Applied Psychology/Institute of Brain and Behavior Amsterdam, Vrije Universiteit Amsterdam, Amsterdam, Netherlands

**Keywords:** sport personality traits, lexical study, HEXACO, Big Five, sport and leisure activities

In the original article, there were errors in Tables 4 and 5 (and in Tables S4.1 through S4.6 in the Supplemental File) as published. Instead of using the natural logarithm (LN) to transform the odd ratios into Cohen's *d*, the common logarithm (LOG) was used. Consequently, all estimates of Cohen's *d* in Tables 4 and 5 (and Supplemental Tables S4.1 through S4.6) are underestimates of the true values. The corrected Tables 4 and 5 and the Supplementary Tables S4.1 through S4.6 to the original article has been updated.

**Table 4:** Logistic regression of the most practiced sports and leisure activities on background and sport personality traits (*N* = 555).

	**Fitness (*n* = 149)**			**Running (*n* = 131)**			**Soccer (*n* = 122)**		
	**Odds**	**95% CI**	***d***	**Odds**	**95% CI**	***d***	**Odds**	**95% CI**	***d***
**Background**									
1. Gender (0 = F, 1 = M)	0.88	(0.59–1.32)	−0.07	1.61^*^	(1.04–2.48)	0.26	11.91^**^	(6.17–23.01)	1.37
2. Age	0.98^**^	(0.96–0.99)	−0.01	0.98^*^	(0.97–1.00)	−0.01	0.99	(0.97–1.01)	−0.01
3. Education (1 = Lo thru 3 = Hi)	1.20	(0.91–1.58)	0.10	1.15	(0.86–1.53)	0.08	0.48^**^	(0.34–0.67)	−0.41
**Sport personality traits**									
4. Friendly Fairness	1.38	(0.79–2.40)	0.18	0.94	(0.53–1.65)	−0.04	0.90	(0.46–1.75)	−0.06
5. Resilience	1.01	(0.62–1.66)	0.01	0.96	(0.55–1.68)	−0.02	0.69	(0.35–1.37)	−0.20
6. Agility	1.13	(0.57–2.25)	0.07	1.69	(0.82–3.48)	0.29	0.98	(0.43–2.23)	−0.01
7. Drive	1.34	(0.70–2.59)	0.16	2.09^*^	(1.03–4.23)	0.41	1.48	(0.68–3.22)	0.22
8. Perfectionism	0.89	(0.45–1.80)	−0.06	0.51	(0.23–1.12)	−0.37	4.69^**^	(1.92–11.47)	0.85
9. Inventiveness	0.39^**^	(0.20–0.75)	−0.52	1.14	(0.58–2.25)	0.07	1.05	(0.49–2.26)	0.03
Nagelkerke pseudo *R^2^*	6.8%	9.2%	37.1%						
χ^2^	26.71, *df* = 9, *p* < 0.01	35.03, *df* = 9, *p* < 0.01	153.58, *df* = 9, *p* < 0.01						

* *p < 0.05*;

** *p < 0.01*.

**Table 5:** Logistic regression of the most practiced sports and leisure activities on background and sport personality traits (*N* = 555).

	**Solving puzzles (*n* = 118)**			**Swimming (*n* = 103)**			**Tennis (*n* = 98)**		
	**Odds**	**95% CI**	***d***	**Odds**	**95% CI**	***d***	**Odds**	**95% CI**	***d***
**Background**									
1. Gender (0 = F, 1 = M)	0.32^**^	(0.20–0.52)	−0.62	0.26^**^	(0.16–0.43)	−0.75	0.72	(0.45–1.16)	−0.18
2. Age	1.07^**^	(1.05–1.09)	0.04	1.00	(0.98–1.02)	0.00	1.01	(1.00–1.03)	0.01
3. Education (1 = Lo thru 3 = Hi)	0.96	(0.71–1.31)	−0.02	1.08	(0.79–1.48)	0.04	1.43^*^	(1.03–1.98)	0.20
**Sport personality traits**									
4. Friendly Fairness	1.12	(0.58–2.15)	0.06	1.45	(0.76–2.76)	0.20	1.43	(0.75–2.74)	0.20
5. Resilience	0.84	(0.48–1.46)	−0.10	1.10	(0.63–1.93)	0.05	0.61	(0.32–1.16)	−0.28
6. Agility	1.02	(0.46–2.26)	0.01	0.91	(0.41–2.00)	−0.05	4.05^**^	(1.74–9.42)	0.77
7. Drive	0.66	(0.30–1.43)	−0.23	0.91	(0.42–2.01)	−0.05	0.72	(0.32–1.63)	−0.18
8. Perfectionism	1.58	(0.69–3.60)	0.25	0.80	(0.35–1.82)	−0.12	1.50	(0.63–3.55)	0.22
9. Inventiveness	0.82	(0.38–1.75)	−0.11	1.40	(0.67–2.93)	0.19	0.86	(0.41–1.83)	−0.08
Nagelkerke pseudo *R^2^*	22.1%	11.2%	7.9%						
χ^2^	85.13, *df* = 9, *p* < 0.01	39.77, *df* = 9, *p* < 0.01	27.16, *df* = 9, *p* < 0.01						

* *p < 0.05*;

** *p < 0.01*.

**Table S4.1:** Logistic regression of practicing fitness on background, HEXACO personality, and sport personality trait variables (*N* = 449).

	**Fitness (*n* = 125)**					
	**Odds**	**95% CI**	***d***	**Odds**	**95% CI**	***D***
**Background**						
1. Gender (0 = F, 1 = M)	0.78	(0.48–1.26)	−0.14	0.79	(0.48–1.30)	−0.13
2. Age	0.98^*^	(0.97–1.00)	−0.01	0.98^*^	(0.96–1.00)	−0.01
3. Education (1 = Lo thru 3 = Hi)	1.22	(0.89–1.69)	0.11	1.22	(0.88–1.70)	0.11
**Personality**						
4. Honesty-Humility	1.43	(0.84–2.42)	0.20	1.21	(0.68–2.13)	0.11
5. Emotionality	1.15	(0.66–1.98)	0.08	1.05	(0.60–1.85)	0.03
6. Extraversion	1.17	(0.71–1.94)	0.09	1.20	(0.69–2.10)	0.10
7. Agreeableness	0.75	(0.43–1.31)	−0.16	0.81	(0.46–1.43)	−0.12
8. Conscientiousness	0.93	(0.52–1.68)	−0.04	0.84	(0.45–1.59)	−0.10
9. Openness to Experience	0.92	(0.56–1.52)	−0.05	1.10	(0.64–1.88)	0.05
**Sport personality traits**						
10. Friendly Fairness				1.43	(0.75–2.75)	0.20
11. Resilience				0.89	(0.51–1.58)	−0.06
12. Agility				1.32	(0.60–2.90)	0.15
13. Drive				1.45	(0.69–3.07)	0.20
14. Perfectionism				0.92	(0.40–2.09)	−0.05
15. Inventiveness				0.32^**^	(0.15–0.70)	−0.63
Nagelkerke pseudo *R^2^*	4.7%	8.1%				
χ^2^	14.75, *df* = 9, *p* = 0.10	25.97, *df* = 15, *p* = 0.04				

* *p < 0.05*;

** *p < 0.01*.

**Table S4.2:** Logistic regression of practicing running on background, HEXACO personality, and sport personality trait variables (*N* = 449).

	**Running (*n* = 104)**					
	**Odds**	**95% CI**	***d***	**Odds**	**95% CI**	***d***
**Background**						
1. Gender (0 = F, 1 = M)	1.71^*^	(1.01–2.90)	0.30	1.46	(0.84–2.51)	0.21
2. Age	0.98^*^	(0.96–0.99)	−0.01	0.98	(0.96–1.00)	−0.01
3. Education (1 = Lo thru 3 = Hi)	1.20	(0.84–1.71)	0.10	1.30	(0.90–1.87)	0.14
**Personality**						
4. Honesty-Humility	0.86	(0.50–1.48)	−0.08	0.97	(0.54–1.75)	−0.02
5. Emotionality	0.90	(0.50–1.62)	−0.06	0.90	(0.49–1.66)	−0.06
6. Extraversion	1.84^*^	(1.06–3.21)	0.34	1.41	(0.77–2.58)	0.19
7. Agreeableness	0.57	(0.31–1.05)	−0.31	0.55	(0.30–1.04)	−0.33
8. Conscientiousness	0.47^*^	(0.25–0.89)	−0.42	0.47^*^	(0.23–0.94)	−0.42
9. Openness to Experience	0.91	(0.53–1.57)	−0.05	0.82	(0.46–1.47)	−0.11
**Sport personality traits**						
10. Friendly Fairness				1.05	(0.54–2.06)	0.03
11. Resilience				0.94	(0.48–1.82)	−0.03
12. Agility				1.51	(0.66–3.49)	0.23
13. Drive				2.22*^†^*	(0.97–5.08)	0.44
14. Perfectionism				0.69	(0.27–1.73)	−0.20
15. Inventiveness				1.09	(0.47–2.50)	0.05
Nagelkerke pseudo *R^2^*	9.7%	13.2%				
χ^2^	29.89, *df* = 9, *p* < 0.01	41.01, *df* = 15, *p* < 0.01				

* *p < 0.05*;

^**^p < 0.01;

† *p = 0.059*.

**Table S4.3:** Logistic regression of playing soccer on background, HEXACO personality, and sport personality trait variables (*N* = 449).

	**Soccer (*n* = 93)**					
	**Odds**	**95% CI**	***d***	**Odds**	**95% CI**	***D***
**Background**						
1. Gender (0 = F, 1 = M)	15.54^**^	(6.77–35.66)	1.51	15.07^**^	(6.30–36.04)	1.50
2. Age	0.99	(0.97–1.01)	−0.01	1.00	(0.98–1.02)	0.00
3. Education (1 = Lo thru 3 = Hi)	0.52^**^	(0.35–0.77)	−0.36	0.49^**^	(0.32–0.75)	−0.39
**Personality**						
4. Honesty-Humility	0.56	(0.30–1.05)	−0.32	0.66	(0.33–1.35)	−0.23
5. Emotionality	1.04	(0.53–2.06)	0.02	1.08	(0.52–2.24)	0.04
6. Extraversion	1.12	(0.59–2.14)	0.06	1.17	(0.55–2.49)	0.09
7. Agreeableness	1.39	(0.71–2.71)	0.18	1.37	(0.67–2.79)	0.17
8. Conscientiousness	1.42	(0.66–3.03)	0.19	0.86	(0.38–1.98)	−0.08
9. Openness to Experience	0.64	(0.34–1.22)	−0.25	0.47^*^	(0.23–0.96)	−0.42
**Sport personality traits**						
10. Friendly Fairness				1.10	(0.48–2.48)	0.05
11. Resilience				0.64	(0.28–1.45)	−0.25
12. Agility				1.02	(0.39–2.71)	0.01
13. Drive				0.92	(0.34–2.45)	−0.05
14. Perfectionism				9.16^**^	(3.05–27.47)	1.22
15. Inventiveness				0.68	(0.26–1.79)	−0.21
Nagelkerke pseudo *R^2^*	32.7%	39.8%				
χ^2^	105.38, *df* = 9, *p* < 0.01	131.73, *df* = 15, *p* < 0.01				

* *p < 0.05*;

** *p < 0.01*.

**Table S4.4:** Logistic regression of solving puzzles on background, HEXACO personality, and sport personality trait variables (*N* = 449).

	**Solving Puzzles (*n* = 98)**					
	**Odds**	**95% CI**	***d***	**Odds**	**95% CI**	***d***
**Background**						
1. Gender (0 = F, 1 = M)	0.32^**^	(0.18–0.58)	−0.63	0.33^**^	(0.18–0.59)	−0.61
2. Age	1.06^**^	(1.04–1.09)	0.03	1.06^**^	(1.04–1.09)	0.03
3. Education (1 = Lo thru 3 = Hi)	0.92	(0.65–1.31)	−0.05	0.90	(0.63–1.29)	−0.06
**Personality**						
4. Honesty-Humility	1.07	(0.56–2.04)	0.04	1.03	(0.52–2.06)	0.02
5. Emotionality	0.95	(0.49–1.84)	−0.03	0.95	(0.49–1.87)	−0.03
6. Extraversion	0.39^**^	(0.21–0.71)	−0.52	0.41^**^	(0.21–0.81)	−0.49
7. Agreeableness	1.57	(0.81–3.03)	0.25	1.55	(0.79–3.05)	0.24
8. Conscientiousness	1.44	(0.71–2.93)	0.20	1.35	(0.64–2.85)	0.17
9. Openness to Experience	1.52	(0.83–2.79)	0.23	1.51	(0.79–2.87)	0.23
**Sport personality traits**						
10. Friendly Fairness				0.96	(0.44–2.07)	−0.02
11. Resilience				1.07	(0.57–2.02)	0.04
12. Agility				0.77	(0.32–1.87)	−0.14
13. Drive				0.92	(0.39–2.19)	−0.05
14. Perfectionism				1.35	(0.51–3.57)	0.17
15. Inventiveness				1.02	(0.42–2.47)	0.01
Nagelkerke pseudo *R^2^*	22.7%	23.0%				
χ^2^	71.80, *df* = 9, *p* < 0.01	72.53, *df* = 15, *p* < 0.01				

^*^p < 0.05;

** *p < 0.01*.

**Table S4.5:** Logistic regression of practicing swimming on background, HEXACO personality, and sport personality trait variables (*N* = 449).

	**Swimming (*n* = 82)**					
	**Odds**	**95% CI**	***d***	**Odds**	**95% CI**	***d***
**Background**						
1. Gender (0 = F, 1 = M)	0.23^**^	(0.13–0.43)	−0.81	0.24^**^	(0.13–0.45)	−0.79
2. Age	1.01	(0.99–1.03)	0.01	1.00	(0.98–1.03)	0.00
3. Education (1 = Lo thru 3 = Hi)	1.09	(0.76–1.59)	0.05	1.09	(0.75–1.59)	0.05
**Personality**						
4. Honesty-Humility	1.19	(0.64–2.22)	0.10	1.10	(0.57–2.13)	0.05
5. Emotionality	0.93	(0.48–1.79)	−0.04	0.92	(0.48–1.79)	−0.05
6. Extraversion	1.53	(0.83–2.82)	0.23	1.60	(0.82–3.11)	0.26
7. Agreeableness	0.84	(0.43–1.65)	−0.10	0.86	(0.44–1.71)	−0.08
8. Conscientiousness	0.86	(0.43–1.72)	−0.08	0.84	(0.40–1.77)	−0.10
9. Openness to Experience	1.44	(0.79–2.63)	0.20	1.51	(0.80–2.87)	0.23
**Sport personality traits**						
10. Friendly Fairness				0.99	(0.47–2.12)	−0.01
11. Resilience				1.17	(0.62–2.22)	0.09
12. Agility				0.78	(0.32–1.92)	−0.14
13. Drive				0.98	(0.40–2.41)	−0.01
14. Perfectionism				1.02	(0.38–2.76)	0.01
15. Inventiveness				0.88	(0.36–2.15)	−0.07
Nagelkerke pseudo *R^2^*	12.4%	12.7%				
χ^2^	35.41, *df* = 9, *p* < 0.01	36.54, *df* = 15, *p* < 0.01				

^*^p < 0.05;

** *p < 0.01*.

**Table S4.6:** Logistic regression of playing tennis on background, HEXACO personality, and sport personality trait variables (*N* = 449).

	**Tennis (*n* = 79)**					
	**Odds**	**95% CI**	***d***	**Odds**	**95% CI**	***d***
**Background**						
1. Gender (0 = F, 1 = M)	0.78	(0.44–1.37)	−0.14	0.69	(0.38–1.24)	−0.20
2. Age	1.00	(0.98–1.02)	0.00	1.01	(0.99–1.03)	0.01
3. Education (1 = Lo thru 3 = Hi)	1.53^*^	(1.04–2.25)	0.22	1.54^*^	(1.03–2.30)	0.24
**Personality**						
4. Honesty-Humility	0.99	(0.53–1.82)	−0.01	1.23	(0.63–2.39)	0.11
5. Emotionality	0.88	(0.46–1.67)	−0.07	0.88	(0.45–1.74)	−0.07
6. Extraversion	0.99	(0.55–1.79)	−0.01	0.88	(0.46–1.71)	−0.07
7. Agreeableness	0.87	(0.45–1.66)	−0.08	0.72	(0.37–1.42)	−0.18
8. Conscientiousness	1.33	(0.67–2.65)	0.16	1.35	(0.63–2.87)	0.17
9. Openness to Experience	0.84	(0.47–1.53)	−0.10	0.71	(0.37–1.36)	−0.19
**Sport personality traits**						
10. Friendly Fairness				1.87	(0.85–4.13)	0.35
11. Resilience				0.57	(0.27–1.20)	−0.31
12. Agility				5.29^**^	(1.98–14.18)	0.92
13. Drive				0.48	(0.18–1.27)	−0.40
14. Perfectionism				1.25	(0.45–3.46)	0.12
15. Inventiveness				0.98	(0.39–2.46)	−0.01
Nagelkerke pseudo *R^2^*	3.0%	9.5%				
χ^2^	8.16, *df* = 9, *p* = 0.52	26.70, *df* = 15, *p* = 0.03				

* *p < 0.05*;

** *p < 0.01*.

The author apologizes for these errors and states that they do not change the scientific conclusions of the article in any way. The original article has been updated.

