# Supplementary material for: The Main Dimensions of Sport Personality Traits: A Lexical Approach
Source: Front Psychol. 2020 Sep 23;11:2211. doi: 10.3389/fpsyg.2020.02211 (PMC7538606; doi:10.3389/fpsyg.2020.02211)
Supplement: Supplementary file 1 [file Data_Sheet_1.pdf]

Supplemental File

Table S1.

The sport personality adjectives, descriptives of phases 2 and 3, and PCA loadings on the first six principal components of the ipsatized sport personality trait adjectives

English (translation)	Dutch (original)	M_{proto}	SD_{proto}	M_{rating}	SD_{rating}	PC1	PC2	PC3	PC4	PC5	PC6
good-humoured	goedgehumeurd	3.29	1.27	3.34	.65	**.56**	.14	-.09	.04	-.20	-.05
kind	vriendelijk	3.57	1.45	3.37	.61	**.55**	-.08	-.09	-.09	-.09	-.01
friendly	vriendschappelijk	3.93	1.07	3.37	.63	**.55**	.00	-.12	-.09	-.14	-.02
social	sociaal	3.21	1.48	3.31	.66	**.52**	.00	-.12	-.04	-.07	.04
fair	fair	3.71	0.91	3.32	.69	**.52**	-.01	-.12	-.09	-.02	.06
tolerant	tolerant	3.14	1.35	3.28	.65	**.52**	-.03	-.15	-.15	-.04	.10
very hard	snoeihard	3.29	1.27	2.20	.87	**-.51**	.17	.03	-.03	-.16	-.12
merciless	genadeloos	3.21	1.25	2.29	.88	**-.51**	.10	.03	-.01	-.12	-.01
solidair	solidair	3.00	1.04	3.28	.61	**.51**	.00	-.13	-.14	-.13	-.05
hard as nails	spijkerhard	3.29	1.33	2.23	.87	**-.50**	.23	-.07	-.02	-.14	-.11

rough	ruig	3.00	1.04	2.25	.83	**-.49**	.06	-.09	-.03	-.21	-.04
destructive	verwoestend	3.00	1.11	2.19	.86	**-.49**	.02	-.05	-.06	-.15	-.01
helpful	hulpvaardig	3.07	1.33	3.38	.66	**.49**	-.06	-.10	-.07	-.10	.06
decent	fatsoenlijk	3.14	1.17	3.31	.66	**.47**	-.02	-.07	-.13	.04	-.07
hard	hard	3.86	0.95	2.43	.82	**-.47**	.21	-.04	.00	-.12	-.03
hard as stone	bikkelhard	4.36	0.63	2.28	.88	**-.47**	.21	-.04	.01	-.14	-.13
attentive	attent	3.14	1.10	3.28	.60	**.47**	.05	-.13	-.06	.06	.03
rock-hard	keihard	3.71	1.07	2.32	.86	**-.46**	.24	-.07	-.06	-.15	-.11
just	correct	3.00	1.18	3.27	.63	**.45**	.00	-.14	-.15	.13	-.04
hostile	vijandig	3.00	1.24	2.08	.82	**-.44**	-.14	-.19	-.17	-.09	-.05
venomous	venijnig	3.07	1.00	2.29	.84	**-.44**	.01	-.13	-.04	-.16	-.02
brutish	grof	3.00	1.30	2.03	.84	**-.44**	-.06	-.20	-.16	-.17	-.17
enraged	woedend	3.00	1.47	2.02	.84	**-.44**	-.18	-.24	-.08	-.11	-.19
honest	eerlijk	3.07	1.49	3.39	.68	**.44**	.02	-.11	-.13	.02	-.03
cheerful	opgewekt	3.64	1.28	3.30	.69	**.44**	.18	-.04	.19	-.20	-.04
merry	vrolijk	3.71	1.38	3.26	.61	**.43**	.14	-.03	.04	-.28	-.05

co-operative	coöperatief	3.36	1.22	3.15	.63	**.43**	.10	-.12	-.07	.04	.05
harsh	hardhandig	3.14	1.17	2.25	.83	**-.43**	.04	.01	-.08	-.20	-.11
antagonistic	vijandelijk	3.14	1.29	2.14	.84	**-.43**	-.11	-.15	-.13	-.16	-.11
harmonious	harmonieus	3.36	1.34	3.08	.59	**.43**	-.05	.11	-.09	.03	-.04
ruthless	meedogenloos	3.36	1.22	2.29	.86	-.42	.16	.00	-.03	-.17	.00
fraternal	broederlijk	3.00	1.04	3.12	.62	.42	.03	-.14	-.04	-.10	.03
amicable	collegiaal	3.07	1.21	3.37	.63	.42	-.04	-.08	-.13	-.10	.04
aggressive	agressief	4.00	1.11	2.08	.84	-.42	-.04	-.21	-.05	-.11	-.11
quiet	rustig	3.43	1.09	3.28	.70	.42	.06	-.09	-.37	.11	-.04
sympathetic	sympathiek	3.36	1.34	3.22	.58	.42	.03	-.03	-.16	-.16	-.01
patient	geduldig	3.50	1.09	3.25	.69	.41	.07	.00	-.32	-.08	-.12
calm	kalm	3.57	0.76	3.24	.66	.41	.12	-.06	-.36	.13	-.03
irascible	driftig	3.00	1.18	2.21	.83	-.41	-.07	-.22	-.04	-.15	-.11
worked up	opgefokt	3.36	1.15	2.18	.82	-.40	-.28	-.20	-.02	-.12	-.16
beastly	beestachtig	3.00	1.04	2.00	.86	-.40	.04	-.08	-.01	-.17	-.05
dangerous	gevaarlijk	3.36	1.28	2.20	.83	-.40	-.02	-.05	-.09	-.18	.01

furious	furieus	3.00	1.24	2.28	.81	-.40	.03	-.10	.01	-.18	.00
hotheaded	heetgebakerd	3.36	1.01	2.15	.85	-.40	-.20	-.18	.06	-.14	-.09
wild	wild	3.50	1.16	2.29	.77	-.39	-.02	-.02	.02	-.24	-.05
sportive	sportief	4.57	0.51	3.14	.72	.39	.16	.08	.13	-.03	-.07
dominant	dominant	3.29	0.99	2.45	.84	-.38	.10	-.15	.07	.06	.07
relaxed	relaxed	3.50	0.76	3.08	.68	.38	.30	.02	-.20	-.09	.04
provocative	provocerend	3.07	1.27	2.24	.82	-.38	-.04	-.15	-.06	-.14	.02
spontaneous	spontaan	3.07	1.38	3.14	.67	.37	.02	.00	.19	-.20	.13
macho	macho	3.21	0.89	2.06	.86	-.37	.05	-.09	-.02	-.15	-.09
selfish	egoïstisch	3.21	1.12	2.34	.83	-.37	-.16	-.17	-.13	-.04	-.08
happy	blij	3.43	1.16	3.22	.67	.37	.09	.05	.27	-.20	-.04
wise	verstandig	3.36	1.08	3.23	.57	.36	.06	-.03	-.23	.13	-.11
explosive	explosief	4.14	0.86	2.52	.85	-.36	.04	.14	.17	-.18	.04
complacent	zelfingenomen	3.14	1.29	2.41	.76	-.36	-.04	-.03	-.10	-.09	-.01
busybodying	uitsloverig	3.21	1.42	2.26	.86	-.36	-.06	-.03	.06	-.07	-.09
extreme	extreem	3.21	1.19	2.32	.83	-.36	.02	.04	.02	-.16	-.03

responsible	verantwoordelijk	3.29	1.27	3.30	.61	.36	-.01	-.17	.01	.19	.02
at ease	ontspannen	3.86	0.77	3.14	.65	.36	.31	.09	-.15	-.12	-.02
out of control	onbeheerst	3.07	1.14	2.29	.76	-.35	-.26	-.12	-.17	-.30	-.10
flexible	flexibel	3.07	1.07	3.10	.66	.35	.15	.13	-.02	-.06	-.01
optimistic	optimistisch	3.36	1.01	3.22	.67	.35	.25	.00	.14	-.02	-.06
self-possessed	beheerst	3.86	0.66	3.18	.60	.35	.10	-.01	-.28	.26	-.13
reliable	betrouwbaar	3.29	0.99	3.37	.64	.34	.12	-.10	-.11	.13	-.10
communicative	communicatief	3.36	1.22	3.14	.70	.33	.08	-.10	.08	-.06	.15
reckless	roekeloos	3.50	1.22	2.23	.77	-.32	-.08	-.06	-.10	-.23	.00
brash	brutaal	3.00	0.96	2.32	.81	-.32	.03	-.14	.09	-.14	.16
shrewd	uitgekookt	3.14	1.17	2.50	.84	-.32	.03	-.16	-.13	.04	.21
attacking	aanvallend	4.29	0.91	2.73	.82	-.32	.10	-.04	.21	.00	.13
aspiring	streberig	3.00	1.47	2.43	.84	-.32	-.10	.03	.10	.05	-.16
fierce	fel	4.14	1.03	2.59	.82	-.32	.10	-.09	.27	-.04	.01
heroic	heroïsch	3.14	1.10	2.52	.74	-.32	.02	.09	.07	-.20	.15
docile	volgzaam	3.07	1.33	2.90	.62	.32	-.26	-.06	-.04	-.05	-.24

underhanded	slinks	3.00	1.18	2.43	.82	-.32	-.05	-.13	-.13	-.06	.13
playful	speels	3.57	1.02	3.00	.66	.31	.02	-.02	.03	-.27	.21
wary	behoedzaam	3.21	1.19	3.16	.63	.31	-.18	-.19	-.30	.10	-.17
egocentric	egocentrisch	3.00	1.11	2.32	.79	-.31	-.13	-.09	-.12	-.04	-.09
unsportsmanlike	onsportief	3.57	1.65	2.18	.85	-.31	-.28	-.17	-.25	-.10	-.09
tactful	tactvol	3.14	1.51	3.15	.62	.31	.01	-.02	-.16	.17	.09
heated	verhit	3.36	1.34	2.46	.82	-.31	-.22	-.14	.08	-.21	-.15
adult	volwassen	3.57	1.22	3.22	.59	.30	.06	-.10	-.16	.04	-.13
invincible	onoverwinlijk	3.29	1.49	2.51	.78	-.29	.16	.17	-.05	-.07	.12
risky	riskant	3.29	1.27	2.39	.76	-.28	.04	-.06	-.05	-.21	.08
perceptive	opmerkzaam	3.00	1.36	3.21	.64	.28	.01	-.11	-.01	.24	-.03
virtuous	eerzaam	3.00	1.18	3.06	.61	.28	-.09	-.04	-.11	.09	.04
observant	oplettend	3.86	0.77	3.26	.65	.28	.09	-.08	-.04	.19	-.10
bold	stoer	3.07	1.21	2.60	.75	-.27	.20	.11	.03	-.14	.06
vehement	vlammend	3.50	1.16	2.57	.75	-.27	.05	.12	.20	-.15	.10
agitated	geprikkeld	3.07	1.27	2.54	.79	-.27	-.26	-.07	-.07	-.02	-.14

solid	solide	3.00	1.04	3.08	.54	.26	.14	-.05	-.04	.15	-.08
valiant	heldhaftig	3.07	1.00	2.60	.75	-.25	.08	.10	.10	-.23	.17
eager to learn	leergierig	3.79	0.80	3.24	.70	.25	.05	-.01	.09	.13	-.01
incalculable	onberekenbaar	3.07	1.27	2.41	.78	-.25	-.12	-.15	-.10	-.21	.04
participatory	deelnemend	3.43	1.28	3.14	.63	.24	.15	-.01	.20	.10	-.01
encouraging	aanmoedigend	3.50	1.16	3.18	.67	.24	.02	-.04	.18	.01	.06
full of character	karaktervol	3.21	1.19	3.15	.59	.24	.11	-.09	.05	-.05	.06
cold-blooded	koelbloedig	3.43	1.28	2.70	.76	-.23	.20	-.05	-.07	.09	.13
male	mannelijk	3.14	1.35	2.59	.85	-.23	.23	-.08	-.01	-.12	.02
undaunted	onverschrokken	3.07	1.21	2.70	.75	-.22	.20	.09	.03	-.04	.11
reactive	reactief	3.14	1.17	2.96	.59	.22	-.09	.02	-.09	.04	-.14
refined	verfijnd	3.21	1.25	2.88	.64	.21	-.04	.18	-.11	.16	.11
honorable	eervol	3.43	1.09	3.06	.65	.19	.00	.00	.05	.12	.13
constructive	constructief	3.07	1.00	3.05	.62	.18	.15	-.11	-.04	.16	.08
conscious	bewust	3.43	0.94	3.23	.61	.17	.12	-.05	-.03	.14	.00
nervous	nerveus	3.29	0.91	2.59	.78	.00	**-.64**	-.07	-.08	-.03	-.14

uncertain	onzeker	3.29	1.38	2.75	.83	.14	**-.64**	-.18	-.23	-.07	-.16
stressed	gestrest	3.14	0.95	2.47	.81	-.12	**-.63**	-.10	-.08	.02	-.07
jittery	zenuwachtig	3.50	1.09	2.63	.81	-.06	**-.62**	-.11	-.09	-.05	-.19
hesitating	twijfelend	3.00	1.24	2.75	.75	.16	**-.61**	-.12	-.20	-.09	-.17
strained	gespannen	3.64	1.01	2.68	.78	-.08	**-.58**	-.16	.01	.02	-.08
spasmodic	verkrampt	3.07	1.54	2.60	.78	-.04	**-.55**	-.18	-.20	-.05	-.10
tired	vermoeid	3.29	1.38	2.90	.83	.07	**-.54**	-.38	-.19	-.06	-.04
exhausted	uitgeput	3.08	1.19	2.81	.83	.04	**-.53**	-.40	-.19	-.06	-.05
weary	afgemat	3.57	0.94	2.75	.83	.05	**-.51**	-.33	-.16	-.07	.02
worn-out	afgepeigerd	3.57	1.16	2.77	.88	-.02	**-.51**	-.30	-.18	.01	-.05
defeated	verslagen	3.14	1.29	2.51	.72	-.03	**-.50**	-.13	-.27	-.10	-.07
frustrated	gefrustreerd	3.43	1.28	2.42	.83	-.17	**-.49**	-.20	-.12	-.04	-.16
clumsy	stuntelig	3.21	1.42	2.68	.87	.10	**-.49**	-.32	-.29	-.13	-.12
self-assured	zelfverzekerd	4.21	0.89	2.99	.70	.01	**.48**	.05	.05	-.02	.03
run-down	oververmoeid	3.07	1.49	2.77	.83	-.02	**-.47**	-.25	-.25	-.06	-.01
restless	rusteloos	3.00	0.88	2.63	.74	-.17	**-.46**	-.21	-.03	-.11	-.04

injured	geblesseerd	3.43	1.34	2.56	.82	-.03	**-.46**	-.19	-.11	.00	-.08
inconsistent	wisselvallig	3.64	1.15	2.73	.70	-.03	**-.45**	-.25	-.12	-.18	.04
battle-weary	moegestreden	3.14	1.23	2.75	.79	.05	**-.44**	-.29	-.21	-.05	.02
stiff	stijf	3.07	1.27	2.80	.88	.05	**-.44**	-.39	-.30	-.05	-.04
forced	geforceerd	3.07	1.21	2.59	.76	-.22	**-.43**	-.13	-.14	-.14	-.10
slow	langzaam	3.07	1.33	2.82	.81	.16	**-.43**	-.37	-.34	-.10	-.14
demotivated	gedemotiveerd	3.00	1.57	2.40	.82	-.03	**-.42**	-.18	-.37	-.11	-.01
amateurish	amateuristisch	3.43	1.09	2.82	.76	.18	**-.41**	-.29	-.26	-.18	-.14
hurried	gehaast	3.00	1.11	2.60	.74	-.20	**-.41**	-.04	.07	-.12	-.12
confident	zelfzeker	3.21	1.31	2.96	.68	.05	**.40**	.08	.07	.04	.01
sloppy	slordig	3.14	1.35	2.48	.74	-.03	**-.40**	-.21	-.18	-.31	.03
cautious	voorzichtig	3.50	1.02	3.15	.70	.37	**-.39**	-.17	-.28	.07	-.23
restrained	terughoudend	3.07	1.14	2.88	.73	.20	**-.38**	-.18	-.33	.06	-.14
certain	zeker	3.64	1.34	3.00	.67	-.02	.37	.08	.04	.11	.04
risk-averse	risicomijdend	3.07	1.27	3.09	.77	.32	-.36	-.21	-.28	.05	-.16
undisciplined	ongedisciplineerd	3.14	1.46	2.30	.79	-.05	-.35	-.09	-.28	-.29	-.02

emotional	emotioneel	3.29	1.38	2.78	.80	.07	-.33	-.05	.10	-.09	.05
strong as a bear	beresterk	3.50	1.09	2.48	.79	-.18	.33	.27	-.02	-.12	-.04
stable	stabiel	3.29	1.33	3.11	.59	.29	.32	-.01	-.14	.09	-.13
firm	vastberaden	3.93	0.83	3.10	.63	.05	.31	-.02	.16	.17	-.16
strong	sterk	3.93	1.07	2.86	.72	-.07	.30	.28	.11	-.03	-.05
leading	leidinggevend	3.14	1.10	2.82	.85	-.10	.29	-.12	.00	.17	.21
determined	vastbesloten	3.57	1.16	3.08	.63	.08	.29	-.08	.19	.13	-.17
leaderlike	leidend	3.50	0.76	2.85	.82	-.10	.29	-.07	.00	.17	.25
indestructible	onverwoestbaar	3.00	1.18	2.60	.77	-.23	.28	.24	.03	-.08	-.05
gutsy	lef	3.43	1.40	2.89	.72	-.15	.28	.08	.19	-.07	.13
resolute	besluitvaardig	3.29	1.07	3.10	.63	.10	.28	-.07	.04	.11	.11
decisive	daadkrachtig	3.79	0.80	3.05	.64	.03	.27	.04	.26	.14	.01
self-conscious	zelfbewust	3.64	0.84	3.05	.65	.09	.26	-.02	.07	.07	-.10
active	actief	4.50	0.85	3.12	.68	.06	.26	.19	.25	.02	-.14
powerful	krachtig	4.14	1.10	2.86	.67	-.03	.26	.21	.12	.05	-.06
convincing	overtuigend	3.79	1.05	3.01	.63	.11	.24	.04	.16	.06	.01

directed	gericht	3.79	0.80	3.16	.62	.16	.23	-.05	.02	.22	-.18
conclusive	beslissend	3.57	1.09	2.96	.65	-.04	.23	.00	-.02	.04	.18
progressive	vooruitstrevend	3.00	1.36	2.96	.66	.07	.23	.11	.12	.03	.20
tenacious	vasthoudend	3.57	1.09	3.12	.65	.09	.22	-.04	.17	.11	-.21
steadfast	standvastig	3.07	1.33	3.05	.62	.19	.21	-.07	.07	.21	-.05
enterprising	ondernemend	3.29	1.27	3.00	.60	.13	.21	.00	.16	.00	.11
sturdy	stevig	3.07	1.14	2.85	.65	-.03	.19	-.02	-.02	-.02	-.08
quick-witted	slagvaardig	3.14	1.29	2.97	.64	.00	.18	.06	.06	.14	.05
unpredictable	onvoorspelbaar	3.57	1.09	2.62	.73	-.13	-.18	-.16	-.06	-.16	.08
courageous	moedig	3.57	0.94	2.91	.63	.08	.17	.12	.10	-.06	.10
ready to start	startklaar	3.07	1.44	3.06	.61	.15	.17	.06	.13	.03	-.05
self-reliant	zelfstandig	3.43	1.09	3.24	.66	.10	.17	-.06	-.08	.13	-.09
tough	taai	3.14	1.29	2.89	.73	.00	.16	-.10	.15	.01	-.02
imperturbable	onverstoorbaar	3.29	1.44	2.98	.74	.05	.13	-.04	-.01	.12	.00
supple	soepel	3.36	1.39	2.86	.76	.21	.12	**.52**	.02	-.03	-.03
fast	snel	3.43	1.22	2.78	.79	-.09	.14	**.52**	.11	.01	.12

swift as an arrow	pijlsnel	3.14	1.51	2.51	.83	-.16	.14	**.51**	.04	-.04	.01
quick	vlug	3.57	1.22	2.77	.74	.00	.09	**.51**	.04	.02	.09
lithe	lenig	3.79	1.12	2.65	.83	.04	.07	**.50**	-.01	-.09	.02
graceful	gracieus	3.29	1.14	2.66	.70	.10	.02	**.50**	-.09	-.05	.06
mobile	beweeglijk	4.14	0.53	2.90	.74	.12	.09	**.50**	.14	-.06	-.03
lightning-fast	bliksemsnel	3.29	1.27	2.50	.84	-.27	.08	**.49**	.04	.00	.03
acrobatic	acrobatisch	3.36	1.55	2.39	.83	-.06	.05	**.45**	-.01	-.06	.11
sluggish	traag	3.07	1.38	2.75	.87	.18	-.43	**-.44**	-.36	-.12	-.16
trained	getraind	4.00	1.24	2.74	.80	-.13	.21	**.41**	.11	.07	-.08
dynamic	dynamisch	3.86	0.86	2.92	.66	.07	.12	**.40**	.21	.00	-.02
inexhaustible	onuitputbaar	3.00	1.18	2.61	.84	-.13	.15	**.39**	.06	-.10	-.02
maneuverable	wendbaar	3.71	1.33	2.90	.66	.19	.10	**.39**	.01	-.03	.04
fluent	vloeiend	3.00	1.36	2.81	.67	.09	.03	**.37**	.05	.07	.03
fleet-footed	lichtvoetig	3.21	1.25	2.68	.74	.09	-.07	**.37**	-.07	-.07	.07
talented	talentvol	3.86	1.17	2.77	.72	.05	.05	**.37**	.01	.19	.23
agile	behendig	4.43	0.76	2.90	.70	.05	.22	**.37**	.09	.17	.16

effortless	moeiteloos	3.36	1.15	2.77	.68	.04	.24	**.36**	-.06	.07	.14
tireless	onvermoeibaar	3.79	1.05	2.74	.78	-.10	.28	**.35**	.02	-.09	-.06
elegant	elegant	3.29	0.83	2.80	.68	.14	.02	**.35**	-.08	.06	.06
excellent	excellent	3.36	1.08	2.68	.73	-.14	.12	**.35**	.00	.07	.22
adept	vlot	3.43	1.02	2.94	.66	.14	.09	**.34**	.20	.07	.04
flashy	flitsend	3.00	1.18	2.63	.72	-.16	.01	**.34**	.10	-.05	.12
vital	vitaal	3.79	1.31	2.96	.71	.15	.31	**.34**	.17	-.01	-.05
cast-iron	ijzersterk	3.71	0.99	2.64	.78	-.21	.30	**.33**	-.03	-.10	-.07
energetic	energiek	4.57	0.51	3.03	.72	.07	.30	**.32**	.28	-.02	-.09
toned	afgetraind	3.71	1.44	2.44	.82	-.18	.07	**.30**	.04	-.03	-.02
resilient	veerkrachtig	3.57	1.28	2.95	.64	.19	.15	.29	.11	.01	.08
muscular	gespierd	3.36	1.22	2.46	.82	-.23	.26	.28	-.07	-.02	-.04
faultless	feilloos	3.14	1.10	2.73	.69	-.14	.05	.27	-.07	.15	.09
physical	fysiek	3.71	1.38	2.81	.73	-.10	.23	.26	.04	-.06	-.12
corporal	lichamelijk	3.29	1.49	2.84	.69	.03	.13	.26	.08	-.08	-.06
professional	professioneel	3.86	0.95	2.92	.73	-.07	.14	.24	.00	.22	.04

sublime	subliem	3.43	1.09	2.69	.72	-.08	.02	.23	-.04	.09	.22
flawless	foutloos	3.14	1.23	2.69	.68	-.03	.00	.23	-.13	.15	-.05
sure	trefzeker	3.79	1.19	2.88	.70	-.09	.20	.21	.07	.15	.18
conditioned	geconditioneerd	3.07	1.27	2.86	.65	.00	.00	.20	-.02	.09	-.12
gifted	begaafd	3.64	1.15	2.85	.67	.02	.11	.17	.01	.13	.13
cool	cool	3.00	1.11	2.75	.68	-.08	.13	.16	-.06	-.08	.10
magnanimous	grootmoedig	3.00	1.04	2.86	.71	.07	-.01	-.15	-.03	-.03	.07
self-satisfied	zelfvoldaan	3.71	1.14	2.79	.70	-.07	.07	.08	.08	-.02	-.06
fanatical	fanatiek	4.86	0.36	2.95	.86	-.25	.14	.07	**.47**	.02	-.17
unmotivated	ongemotiveerd	3.21	1.53	2.36	.83	.03	-.40	-.20	**-.44**	-.12	-.03
driven	gedreven	4.36	0.50	3.10	.75	-.06	.14	.09	**.44**	.08	-.18
enthusiastic	enthousiast	4.43	0.65	3.24	.69	.23	.13	-.03	**.44**	-.06	-.09
ardent	passioneel	3.29	1.49	2.92	.73	-.05	-.02	.14	**.43**	.01	.08
passive	passief	3.07	1.33	2.72	.79	.05	-.38	-.17	**-.43**	-.12	-.09
pugnacious	strijdlustig	4.43	0.51	2.90	.79	-.10	.21	.00	**.42**	.02	-.12
assertive	strijdbaar	3.93	1.00	2.92	.75	-.08	.23	-.08	**.42**	.05	.00

passionate	gepassioneerd	4.43	0.51	3.01	.76	.00	.09	.04	**.41**	-.01	-.01
eager	gretig	3.29	1.27	2.83	.76	-.11	.06	.04	**.40**	.02	.02
animated	bevlogen	4.14	0.95	2.95	.74	-.06	.03	.00	**.38**	.07	.06
temperamentful	temperamentvol	4.00	1.04	2.81	.74	-.13	.01	-.04	**.37**	-.07	.00
combative	strijdvaardig	3.86	1.17	2.94	.76	-.13	.22	-.04	**.37**	-.02	-.04
fiery	vurig	4.00	0.78	2.65	.79	-.22	.01	.06	**.35**	-.08	-.03
motivated	gemotiveerd	4.50	0.52	3.23	.69	.16	.19	.03	**.35**	.15	-.19
intense	intens	3.64	0.93	2.98	.71	.07	.06	.08	**.34**	.00	-.05
truculent	vechtlustig	4.14	0.86	2.77	.82	-.22	.12	-.05	**.33**	-.08	-.08
hot-blooded	hartstochtelijk	3.29	1.20	2.87	.73	-.07	-.01	.09	**.33**	-.16	.02
strong-willed	wilskrachtig	3.57	1.02	3.11	.65	.08	.20	.03	**.32**	.09	-.13
exuberant	uitbundig	3.71	1.14	2.85	.76	-.03	.04	-.01	**.32**	-.22	.09
zealous	ijverig	3.36	1.08	3.14	.68	.18	.17	.03	**.32**	.18	-.13
impassioned	geestdriftig	3.29	1.20	2.91	.70	-.03	.03	-.04	**.31**	-.06	-.01
persevering	volhardend	3.86	1.03	3.08	.71	.09	.23	-.07	**.31**	.07	-.13
inspirited	bezield	3.14	1.23	2.97	.68	.12	.01	.01	**.31**	.01	.03

elated	opgetogen	3.14	1.51	3.05	.63	.28	.05	.03	**.30**	-.16	-.01
nonchalant	nonchalant	3.36	1.22	2.64	.74	-.07	-.30	-.10	**-.30**	-.28	.03
ambitious	ambitieus	3.64	1.08	2.90	.77	-.13	.22	.08	.29	.14	-.09
predictable	voorspelbaar	3.43	1.09	2.87	.61	.12	-.27	-.05	-.29	-.05	-.25
defensive	defensief	3.71	1.20	2.81	.69	.00	-.21	-.16	-.28	.05	-.12
competitive	competitief	4.57	0.85	2.92	.81	-.14	.15	.00	.27	.06	-.02
racy	pittig	3.00	1.18	2.84	.66	-.14	.13	.08	.26	.01	.00
lively	levendig	3.93	1.14	3.09	.64	.16	.09	.17	.25	-.08	.01
inspired	geïnspireerd	3.43	1.40	3.08	.64	.13	.11	.05	.25	.00	.10
dedicated	toegewijd	3.79	0.80	3.20	.65	.20	.07	-.01	.25	.03	-.17
strijdzuchtig	strijdzuchtig	3.43	1.45	2.73	.77	-.23	.21	-.04	.25	-.06	-.06
triumphant	triomfantelijk	3.64	1.15	2.72	.73	-.09	.04	.02	.24	-.13	.09
euphoric	uitgelaten	3.43	1.16	2.86	.69	.11	-.06	-.09	.24	-.18	.08
expressive	expressief	3.07	1.21	2.87	.69	.05	-.04	.08	.22	-.10	.16
proud	trots	3.79	1.12	2.98	.68	.09	.02	.04	.21	-.07	-.01
guarding	verdedigend	3.79	1.12	2.84	.62	.13	-.18	-.08	-.19	.05	-.07

present	aanwezig	3.36	1.28	3.02	.69	-.06	.10	-.09	.19	.07	.01
brave	dapper	3.29	1.07	2.92	.65	.07	.13	.06	.19	-.09	.05
business-like	zakelijk	3.07	1.27	2.76	.74	-.12	.00	-.10	-.17	.15	.05
independent	onafhankelijk	3.14	0.95	3.13	.68	.06	.10	-.05	-.17	.07	-.07
automatic	automatisch	3.14	1.17	2.83	.60	.13	-.12	.04	-.16	.07	-.08
meticulous	secuur	3.36	1.08	3.18	.64	.19	-.01	-.03	-.05	**.46**	-.03
accurate	accuraat	3.00	1.11	3.11	.66	.21	.09	-.10	-.03	**.44**	.06
perfectionistic	perfectionistisch	3.79	1.48	2.99	.83	.01	-.05	.10	.14	**.43**	.12
thorough	nauwkeurig	3.36	0.93	3.19	.64	.17	.02	.01	-.08	**.40**	-.05
precise	precies	3.21	1.19	3.19	.66	.12	-.02	.05	-.09	**.37**	-.02
careful	zorgvuldig	3.50	1.02	3.22	.61	.27	.07	-.04	-.11	**.36**	-.13
tactical	tactisch	4.43	0.85	3.09	.68	.04	.19	-.01	-.03	**.36**	.23
skilled	vakkundig	3.29	1.20	2.96	.73	-.02	.19	.22	-.02	**.35**	.15
deadly serious	bloedserieus	3.43	1.09	2.94	.78	-.06	.02	-.02	.21	**.35**	-.22
efficient	efficiënt	3.93	1.00	3.10	.64	.10	.17	-.02	-.08	**.35**	.04
capable	deskundig	3.07	1.00	2.94	.69	.10	.17	.22	.02	**.35**	.18

systematical	systematisch	3.14	1.10	3.02	.63	.17	.09	-.07	-.13	**.35**	.07
proficient	vaardig	4.07	0.83	2.99	.64	.05	.19	.24	.06	**.34**	.12
well thought-out	weldoordacht	3.07	1.27	3.16	.65	.16	.06	-.14	-.18	**.34**	.07
well considered	doordacht	3.36	0.93	3.11	.67	.13	.10	-.08	-.09	**.33**	.15
specialistic	specialistisch	3.00	1.18	2.80	.71	-.09	.10	.17	.03	**.32**	.22
alert	alert	4.21	0.89	3.20	.66	.04	.02	.01	.08	**.31**	-.02
overconfident	overmoedig	3.64	0.93	2.50	.74	-.19	-.08	-.02	.04	**-.31**	-.03
targeted	doelgericht	4.43	0.51	3.17	.65	.12	.25	-.03	.21	**.30**	-.01
deliberate	weloverwogen	3.50	1.22	3.13	.60	.29	.12	-.12	-.16	.29	-.04
intelligent	intelligent	3.36	1.01	3.16	.60	.17	-.01	-.10	-.12	.29	.18
unrestrained	ongeremd	3.14	1.10	2.57	.77	-.26	-.02	.10	.11	-.28	.00
efficacious	doelmatig	3.71	0.83	3.13	.65	.08	.26	.02	.06	.28	-.04
consistent	consistent	3.07	0.73	3.04	.61	.19	.17	.00	-.10	.27	-.15
disciplined	gedisciplineerd	4.00	1.04	3.21	.70	.22	.19	.01	.00	.26	-.23
skillful	bekwaam	4.00	0.68	2.98	.63	.06	.20	.26	.02	.26	.05
seasoned	geroutineerd	4.00	0.68	2.96	.70	.04	.16	.16	-.01	.26	.09

critical	kritisch	3.14	1.35	3.08	.71	.00	-.13	-.07	.01	.26	-.02
effective	doeltreffend	4.21	0.70	3.02	.64	-.02	.25	.07	.05	.25	.11
impulsive	impulsief	4.00	0.68	2.80	.74	.02	-.11	-.02	.16	-.24	.19
carefree	onbezorgd	3.00	1.41	2.93	.65	.13	.14	-.03	-.23	-.23	.03
purposeful	doelbewust	4.14	0.86	3.15	.65	.06	.21	.04	.07	.23	-.05
productive	productief	3.50	0.76	3.01	.62	.18	.21	.18	.03	.22	.05
vigorous	doortastend	3.00	1.04	3.01	.63	.08	.19	.02	.12	.20	.11
technical	technisch	3.71	0.91	2.85	.71	.02	.13	.17	-.13	.20	.20
competent	competent	3.64	0.93	3.04	.61	.14	.18	.16	.00	.19	.04
experienced	routineus	3.07	0.92	2.90	.66	-.01	-.02	.10	-.13	.18	.01
routine	routinematig	3.00	1.18	2.90	.66	.08	-.10	.11	-.05	.17	-.11
proactive	proactief	3.43	1.40	2.95	.67	.03	.06	.03	.15	.15	.06
full of fantasy	fantasierijk	3.07	1.33	2.96	.69	.14	-.05	.02	.03	-.02	**.44**
creative	creatief	3.57	1.50	3.06	.69	.07	.07	.00	-.01	.04	**.44**
imaginative	fantasievol	3.21	1.25	2.95	.68	.17	.00	.03	.05	-.02	**.42**
inventive	inventief	3.43	1.28	3.01	.64	.11	.12	.01	-.01	.15	**.39**

resourceful	vindingrijk	3.50	1.16	3.06	.65	.15	.13	-.01	-.07	.21	**.38**
innovative	innovatief	3.00	1.36	2.92	.67	.04	.04	.09	.03	.17	**.37**
surprising	verrassend	3.36	1.28	2.91	.65	.05	.03	.17	.04	-.06	**.36**
smart	slim	3.57	1.16	3.11	.66	.14	.16	-.07	-.08	.26	**.30**
serious	serieus	3.79	0.89	3.19	.67	.22	.05	-.05	.08	.30	**-.30**
strategic	strategisch	3.86	0.77	3.01	.71	-.03	.21	-.05	-.03	.26	.28
adroit	virtuoos	3.29	1.49	2.64	.70	-.03	-.07	.24	.04	.01	.25
varied	gevarieerd	3.21	1.25	3.03	.58	.18	-.01	.12	-.05	.08	.24
concentrated	geconcentreerd	4.57	0.65	3.24	.65	.07	.08	.03	.09	.16	-.24
sly	sluw	3.50	1.22	2.57	.83	-.21	.04	-.18	-.13	.05	.23
instinctive	instinctief	3.21	1.25	3.00	.68	.15	.01	-.01	.03	.03	.23
strenuous	inspannend	3.50	1.16	3.05	.63	.20	.02	.00	.21	-.05	-.23
charismatic	charismatisch	3.36	0.93	2.82	.71	.06	.04	.14	.06	-.01	.22
versatile	veelzijdig	3.64	1.22	2.98	.65	.09	.11	.17	.04	.14	.22
intent	ingespannen	3.71	0.99	3.03	.64	.03	-.10	-.02	.13	.09	-.22
determinative	bepalend	3.14	1.29	2.81	.69	-.13	.15	.05	.17	.05	.22

challenging	uitdagend	3.79	0.97	2.71	.73	-.15	.02	.03	.18	-.18	.21
daring	gedurfd	3.64	1.08	2.76	.70	-.15	.17	.08	.14	-.13	.20
intuitive	intuïtief	3.36	1.28	3.06	.65	.12	.00	.02	.06	.02	.20
hardy	flink	3.00	1.30	2.94	.63	.04	.15	.07	.10	-.06	-.18
attractive	attractief	3.14	1.23	2.81	.65	.09	.05	.14	.02	-.08	.16
opportunistic	opportunistisch	3.57	1.02	2.83	.68	-.02	.05	.01	-.03	-.03	.16
offensive	offensief	4.00	1.11	2.81	.69	-.09	.09	.04	.00	.00	.12

Notes: M_{proto}/SD_{proto} = average/standard deviation prototypicality of the adjectives in phase 2; M_{rating}/SD_{rating} = average/standard deviation of the self-ratings on the adjectives in phase 3. The adjectives with bold loadings are also reported in Table 2 of the main document with PC1 = Friendly-Fairness, PC2 = Resilience, PC3 = Agility, PC4 = Drive, PC5 = Perfectionism, and PC6 = Inventiveness.

Table S2.

Description of the factor solutions containing seven through 10 Principal Components (PCs)

# PCs	Description of the content of the Principal Components (PCs)
7:	The seven PCs solution yielded highly similar results as the six PC solution plus an additional 'Courage' seventh component. Its five highest loading adjectives were valiant (heldhaftig; -.35), heroic (heroisch; -.33), self-reliant (zelfstandig; .30), sublime (subliem; -.29), and proactive (proactief; .26). All subsequent absolute loadings of this component were lower than .25.
8:	In the eight PCs solution, components one through five remained virtually unchanged and components six and seven of the seven PC solution moved to components seven and eight of the eight PC solution. The new component six now contained adjectives mainly from the negative pole of the first component of the Friendly Fairness component, e.g., hard as nails (spijkerhard; -.45), very hard (snoeihard; -.43), rock-hard (keihard; -.43), rough (ruig; -.42), and hard as stone (bikkelhard; -.41).
9:	In the nine PCs solution, the variance associated with the new factor in the eight PC solution shifted back to the first component, with components one through six of the nine PC solution virtually indistinguishable from components one through six of the six PC solution. Component seven yielded a mostly new component, which pitted manly (mannelijk; -.41), sly (sluw; -.38), cunning (uitgekookt; -.32), offensive (aanvallend; -.31), strategic (strategisch; -.31), and cool-headed (koelbloedig; -.29) against happy (blij; .31) and concentrated (geconcentreerd; .28). Component eight was highly similar to the 'Courage' component in the previous two solutions, and component nine yielded an 'Experience' component, with highest loading adjectives routine (routinematig; .39), seasoned (geroutineerd; .33), automatic (automatisch; .31), and experienced (routineus; .30).
10:	In the ten PCs solution, components one through six remained virtually unchanged from the previous solution and from the six PC solution. Component seven was similar to the 'Experience' component in the nine PC solution. Component eight was characterized by diverse adjectives related mainly to sly and offensive behaviors, e.g., concentrated (geconcentreerd; .36) versus manly (mannelijk; -.41), sly (sluw; -.34), offensive (offensief; -.32), cunning (uitgekookt; -.32), and attacking (aanvallend; -.30). The highest loading adjectives of component nine were enthousiastic (enthousiast; .36), proactive (proactief; .31), and impulsive (impulsief; .29) versus 'strong as a bear' (beresterk; -.32) and indestructible (onverwoestbaar; -.30). Finally, component ten referred to tenacity , with high loading adjectives such as tenacious (vasthoudend; .38), determined (vastbesloten; .36), strong-willed (wilskrachtig; .34), sturdy (stevig; .30), tough (taai; .30), and hardy (flink; .30).

Table S3.

Correlations between sport personality marker scales and facets of HEXACO personality (N = 449)†

	Friendly Fairness	Resilience	Agility	Drive	Perfect-ionism	Invent-iveness
**Honesty-Humility**						
Sincerity	.30	.11	-.18	-.12	-.07	-.12
Fairness	.31	.08	-.20	-.09	-.07	-.12
Greed Avoidance	.27	.07	-.23	-.12	-.12	-.14
Modesty	.25	.03	-.29	-.19	-.14	-.26
**Emotionality**						
Fearfulness	.11	-.22	-.27	-.29	-.17	-.22
Anxiety	.01	-.23	-.18	-.11	-.10	-.12
Dependence	-.03	-.14	-.09	-.10	-.16	-.13
Sentimentality	.16	-.08	-.10	-.05	-.13	-.08
**Extraversion**						
Social Self-Esteem	.19	.31	.13	.13	.09	.11
Social Boldness	.05	.22	.18	.19	.17	.21
Sociability	-.03	.14	.19	.21	.01	.14
Liveliness	.10	.38	.28	.32	.14	.19
**Agreeableness**						
Forgiveness	.02	.03	.05	.07	.04	.11
Gentleness	.22	.09	-.02	.00	.05	.06
Flexibility	.21	.13	-.08	-.04	-.03	.00
Patience	.22	.15	.06	.01	.15	.10
**Conscientiousness**						
Organization	.05	.13	.00	.02	.06	-.11
Diligence	.04	.27	.18	.22	.19	.13
Perfectionism	.19	.06	-.06	.06	.17	-.04
Prudence	.08	.07	.03	.00	.22	.03
**Openness to Experience**						
Aesthetic Appreciation	.25	.06	-.07	-.03	.04	.06
Inquisitiveness	.12	.14	.10	.09	.18	.18
Creativity	.03	.09	.18	.15	.16	.37
Unconventionality	-.03	-.04	.13	.13	.11	.26
**Interstitial facets**						
Altruism	.35	.05	-.19	-.09	-.11	-.10
Proactivity	.08	.21	.22	.23	.31	.19

† Except for the interstitial facet Proactivity (*N* = 271)

* *p* < .05; ** *p* < .01

Table S4.1.

Logistic regression of practicing fitness on background, HEXACO personality, and sport personality trait variables (N = 449)

	Fitness (n = 125)					
	Odds	95% CI	*d*	Odds	95% CI	*d*
**Background**						
1. Gender (0=F, 1=M)	0.78	(0.48-1.26)	-.14	0.79	(0.48-1.30)	-.13
2. Age	0.98*	(0.97-1.00)	-.01	0.98*	(0.96-1.00)	-.01
3. Education (1=Lo thru 3=Hi)	1.22	(0.89-1.69)	.11	1.22	(0.88-1.70)	.11
**Personality**						
4. Honesty-Humility	1.43	(0.84-2.42)	.20	1.21	(0.68-2.13)	.11
5. Emotionality	1.15	(0.66-1.98)	.08	1.05	(0.60-1.85)	.03
6. Extraversion	1.17	(0.71-1.94)	.09	1.20	(0.69-2.10)	.10
7. Agreeableness	0.75	(0.43-1.31)	-.16	0.81	(0.46-1.43)	-.12
8. Conscientiousness	0.93	(0.52-1.68)	-.04	0.84	(0.45-1.59)	-.10
9. Openness to Experience	0.92	(0.56-1.52)	-.05	1.10	(0.64-1.88)	.05
**Sport personality traits**						
10. Friendly Fairness				1.43	(0.75-2.75)	.20
11. Resilience				0.89	(0.51-1.58)	-.06
12. Agility				1.32	(0.60-2.90)	.15
13. Drive				1.45	(0.69-3.07)	.20
14. Perfectionism				0.92	(0.40-2.09)	-.05
15. Inventiveness				0.32**	(0.15-0.70)	-.63
Nagelkerke pseudo R^2		4.7%			8.1%	
χ^2		14.75, df = 9, p = .10			25.97, df = 15, p = .04	

* p < .05; ** p < .01

Table S4.2.

Logistic regression of practicing running on background, HEXACO personality, and sport personality trait variables (N = 449)

	Running (*n* = 104)					
	Odds	95% CI	*d*	Odds	95% CI	*d*
**Background**						
1. Gender (0=F, 1=M)	1.71*	(1.01-2.90)	.30	1.46	(0.84-2.51)	.21
2. Age	0.98*	(0.96-0.99)	-.01	0.98	(0.96-1.00)	-.01
3. Education (1=Lo thru 3=Hi)	1.20	(0.84-1.71)	.10	1.30	(0.90-1.87)	.14
**Personality**						
4. Honesty-Humility	0.86	(0.50-1.48)	-.08	0.97	(0.54-1.75)	-.02
5. Emotionality	0.90	(0.50-1.62)	-.06	0.90	(0.49-1.66)	-.06
6. Extraversion	1.84*	(1.06-3.21)	.34	1.41	(0.77-2.58)	.19
7. Agreeableness	0.57	(0.31-1.05)	-.31	0.55	(0.30-1.04)	-.33
8. Conscientiousness	0.47*	(0.25-0.89)	-.42	0.47*	(0.23-0.94)	-.42
9. Openness to Experience	0.91	(0.53-1.57)	-.05	0.82	(0.46-1.47)	-.11
**Sport personality traits**						
10. Friendly Fairness				1.05	(0.54-2.06)	.03
11. Resilience				0.94	(0.48-1.82)	-.03
12. Agility				1.51	(0.66-3.49)	.23
13. Drive				2.22†	(0.97-5.08)	.44
14. Perfectionism				0.69	(0.27-1.73)	-.20
15. Inventiveness				1.09	(0.47-2.50)	.05
Nagelkerke pseudo R^2		9.7%			13.2%	
χ^2		29.89, *df* = 9, *p* < .01			41.01, *df* = 15, *p* < .01	

* *p* < .05; ** *p* < .01; † *p* = .059

Table S4.3.

Logistic regression of playing soccer on background, HEXACO personality, and sport personality trait variables (N = 449)

	Soccer (n = 93)					
	Odds	95% CI	*d*	Odds	95% CI	*d*
**Background**						
1. Gender (0=F, 1=M)	15.54**	(6.77-35.66)	1.51	15.07**	(6.30-36.04)	1.50
2. Age	0.99	(0.97-1.01)	-.01	1.00	(0.98-1.02)	.00
3. Education (1=Lo thru 3=Hi)	0.52**	(0.35-0.77)	-.36	0.49**	(0.32-0.75)	-.39
**Personality**						
4. Honesty-Humility	0.56	(0.30-1.05)	-.32	0.66	(0.33-1.35)	-.23
5. Emotionality	1.04	(0.53-2.06)	.02	1.08	(0.52-2.24)	.04
6. Extraversion	1.12	(0.59-2.14)	.06	1.17	(0.55-2.49)	.09
7. Agreeableness	1.39	(0.71-2.71)	.18	1.37	(0.67-2.79)	.17
8. Conscientiousness	1.42	(0.66-3.03)	.19	0.86	(0.38-1.98)	-.08
9. Openness to Experience	0.64	(0.34-1.22)	-.25	0.47*	(0.23-0.96)	-.42
**Sport personality traits**						
10. Friendly Fairness				1.10	(0.48-2.48)	.05
11. Resilience				0.64	(0.28-1.45)	-.25
12. Agility				1.02	(0.39-2.71)	.01
13. Drive				0.92	(0.34-2.45)	-.05
14. Perfectionism				9.16**	(3.05-27.47)	1.22
15. Inventiveness				0.68	(0.26-1.79)	-.21
Nagelkerke pseudo R^2	32.7%			39.8%		
χ^2	105.38, $df = 9$, $p < .01$			131.73, $df = 15$, $p < .01$		

* $p < .05$; ** $p < .01$

Table S4.4.

Logistic regression of solving puzzles on background, HEXACO personality, and sport personality trait variables (N = 449)

	Solving Puzzles (n = 98)					
	Odds	95% CI	*d*	Odds	95% CI	*d*
**Background**						
1. Gender (0=F, 1=M)	0.32**	(0.18-0.58)	-.63	0.33**	(0.18-0.59)	-.61
2. Age	1.06**	(1.04-1.09)	.03	1.06**	(1.04-1.09)	.03
3. Education (1=Lo thru 3=Hi)	0.92	(0.65-1.31)	-.05	0.90	(0.63-1.29)	-.06
**Personality**						
4. Honesty-Humility	1.07	(0.56-2.04)	.04	1.03	(0.52-2.06)	.02
5. Emotionality	0.95	(0.49-1.84)	-.03	0.95	(0.49-1.87)	-.03
6. Extraversion	0.39**	(0.21-0.71)	-.52	0.41**	(0.21-0.81)	-.49
7. Agreeableness	1.57	(0.81-3.03)	.25	1.55	(0.79-3.05)	.24
8. Conscientiousness	1.44	(0.71-2.93)	.20	1.35	(0.64-2.85)	.17
9. Openness to Experience	1.52	(0.83-2.79)	.23	1.51	(0.79-2.87)	.23
**Sport personality traits**						
10. Friendly Fairness				0.96	(0.44-2.07)	-.02
11. Resilience				1.07	(0.57-2.02)	.04
12. Agility				0.77	(0.32-1.87)	-.14
13. Drive				0.92	(0.39-2.19)	-.05
14. Perfectionism				1.35	(0.51-3.57)	.17
15. Inventiveness				1.02	(0.42-2.47)	.01
Nagelkerke pseudo R^2	22.7%			23.0%		
χ^2	71.80, *df* = 9, $p < .01$			72.53, *df* = 15, $p < .01$		

* $p < .05$; ** $p < .01$

Table S4.5.

Logistic regression of practicing swimming on background, HEXACO personality, and sport personality trait variables (N = 449)

	Swimming (n = 82)					
	Odds	95% CI	*d*	Odds	95% CI	*d*
**Background**						
1. Gender (0=F, 1=M)	0.23**	(0.13-0.43)	-.81	0.24**	(0.13-0.45)	-.79
2. Age	1.01	(0.99-1.03)	.01	1.00	(0.98-1.03)	.00
3. Education (1=Lo thru 3=Hi)	1.09	(0.76-1.59)	.05	1.09	(0.75-1.59)	.05
**Personality**						
4. Honesty-Humility	1.19	(0.64-2.22)	.10	1.10	(0.57-2.13)	.05
5. Emotionality	0.93	(0.48-1.79)	-.04	0.92	(0.48-1.79)	-.05
6. Extraversion	1.53	(0.83-2.82)	.23	1.60	(0.82-3.11)	.26
7. Agreeableness	0.84	(0.43-1.65)	-.10	0.86	(0.44-1.71)	-.08
8. Conscientiousness	0.86	(0.43-1.72)	-.08	0.84	(0.40-1.77)	-.10
9. Openness to Experience	1.44	(0.79-2.63)	.20	1.51	(0.80-2.87)	.23
**Sport personality traits**						
10. Friendly Fairness				0.99	(0.47-2.12)	-.01
11. Resilience				1.17	(0.62-2.22)	.09
12. Agility				0.78	(0.32-1.92)	-.14
13. Drive				0.98	(0.40-2.41)	-.01
14. Perfectionism				1.02	(0.38-2.76)	.01
15. Inventiveness				0.88	(0.36-2.15)	-.07
Nagelkerke pseudo R^2	12.4%			12.7%		
χ^2	35.41, $df = 9, p < .01$			36.54, $df = 15, p < .01$		

* $p < .05$; ** $p < .01$

Table S4.6.

Logistic regression of playing tennis on background, HEXACO personality, and sport personality trait variables (N = 449)

	Tennis (n = 79)					
	Odds	95% CI	*d*	Odds	95% CI	*d*
**Background**						
1. Gender (0=F, 1=M)	0.78	(0.44-1.37)	-.14	0.69	(0.38-1.24)	-.20
2. Age	1.00	(0.98-1.02)	.00	1.01	(0.99-1.03)	.01
3. Education (1=Lo thru 3=Hi)	1.53*	(1.04-2.25)	.22	1.54*	(1.03-2.30)	.24
**Personality**						
4. Honesty-Humility	0.99	(0.53-1.82)	-.01	1.23	(0.63-2.39)	.11
5. Emotionality	0.88	(0.46-1.67)	-.07	0.88	(0.45-1.74)	-.07
6. Extraversion	0.99	(0.55-1.79)	-.01	0.88	(0.46-1.71)	-.07
7. Agreeableness	0.87	(0.45-1.66)	-.08	0.72	(0.37-1.42)	-.18
8. Conscientiousness	1.33	(0.67-2.65)	.16	1.35	(0.63-2.87)	.17
9. Openness to Experience	0.84	(0.47-1.53)	-.10	0.71	(0.37-1.36)	-.19
**Sport personality traits**						
10. Friendly Fairness				1.87	(0.85-4.13)	.35
11. Resilience				0.57	(0.27-1.20)	-.31
12. Agility				5.29**	(1.98-14.18)	.92
13. Drive				0.48	(0.18-1.27)	-.40
14. Perfectionism				1.25	(0.45-3.46)	.12
15. Inventiveness				0.98	(0.39-2.46)	-.01
Nagelkerke pseudo R^2	3.0%			9.5%		
χ^2	8.16, df = 9, p = .52			26.70, df = 15, p = .03		

* p < .05; ** p < .01
